# Optimization of PhysicoChemical Parameters for Production of Cytotoxic Secondary Metabolites and Apoptosis Induction Activities in the Culture Extract of a Marine Algal–Derived Endophytic Fungus *Aspergillus* sp.

**DOI:** 10.3389/fphar.2021.542891

**Published:** 2021-04-26

**Authors:** Sidhartha Taritla, Madhuree Kumari, Siya Kamat, Sarita G. Bhat, C. Jayabaskaran

**Affiliations:** ^1^ Department of Biochemistry, Indian Institute of Science, Bangalore, India; ^2^ Department of Biotechnology, Cochin University of Science and Technology, Kochi, India

**Keywords:** marine macroalga, *Sargassum muticum*, endophytic fungi, *Aspergillus* sp., cytotoxic compounds, apoptosis 3

## Abstract

The endophytic fungal community in the marine ecosystem has been demonstrated to be relevant source of novel and pharmacologically active secondary metabolites. The current study focused on the evaluation of cytotoxic and apoptosis induction potential in the culture extracts of endophytic fungi associated with *Sargassum muticum*, a marine brown alga. The cytotoxicity of the four marine endophytes, *Aspergillus* sp., *Nigrospora sphaerica*, *Talaromyces purpureogenus*, and *Talaromyces stipitatus*, was evaluated by the MTT assay on HeLa cells. Further, several physicochemical parameters, including growth curve, culture media, and organic solvents, were optimized for enhanced cytotoxic activity of the selected extract. The *Aspergillus* sp. ethyl acetate extract (ASE) showed maximum cytotoxicity on multiple cancer cell lines. Chemical investigation of the metabolites by gas chromatography–mass spectroscopy (GC-MS) showed the presence of several compounds, including quinoline, indole, 2,4-bis(1,1-dimethylethyl) phenol, and hexadecenoic acid, known to be cytotoxic in ASE. The ASE was then tested for cytotoxicity *in vitro* on a panel of six human cancer cell lines, namely, HeLa (cervical adenocarcinoma), MCF-7 (breast adenocarcinoma), Hep G2 (hepatocellular carcinoma), A-549 (lung carcinoma), A-431 (skin/epidermis carcinoma), and LN-229 (glioblastoma). HeLa cells were most vulnerable to ASE treatment with an IC_50_ value of 24 ± 2 μg/ml. The mechanism of cytotoxicity exhibited by the ASE was further investigated on Hela cells. The results showed that the ASE was capable of inducing apoptosis in HeLa cells through production of reactive oxygen species, depolarization of mitochondrial membrane, and activation of the caspase-3 pathway, which shows a possible activation of the intrinsic apoptosis pathway. It also arrested the HeLa cells at the G2/M phase of the cell cycle, eventually leading to apoptosis. Through this study, we add to the knowledge about the marine algae associated with fungal endophytes and report its potential for purifying specific compounds responsible for cytotoxicity.

## Introduction

Cancer is an immense burden of disease on developing and developed nations, with enormous social and economic impacts. Even in the 21st century, more people die of cancer than a combination of several diseases. The complexity of this disease and overlapping signaling pathways pose a challenge for therapeutic approaches (Alves et al., 2018). More than half of the anticancer drugs commercially available are either direct natural products or their derivatives (Veeresham, 2012). The Indian medicine system continues to prevail on natural sources. In the past, drugs derived from natural sources like medicinal plants have laid an invaluable foundation for the modern day discovery and developments of new drugs (Balunas and Kinghorn, 2005). However, the hurdle lies in evidence-based determination of the pharmacological properties of these drugs and extracts. Hence, scientific validation of these drugs and extracts is a vital part of pharmacology (Choudhury et al., 2016). Till date, the major treatment for cancer has been chemotherapy. There is an urgent need for the development of new anticancer drugs because of increasing resistance of cancer cells to the currently used anticancer drugs, rendering them ineffective. The inevitable side effects of this therapy pose a major hurdle in its application and therapeutic success. Moreover, cancer manifests itself in various forms, making its treatment even more challenging. This clearly unveils an intricate field of research that can give myriads of treatment modalities for the disease (Nurgali et al., 2018). Natural products have therefore been recognized as a promising alternative source in the search for new potent and pharmaceutically effective anticancer compounds.

Recently, the interest in marine microorganisms has grown due to the prospect of getting profuse biomass (Deshmukh et al., 2018; Kamat et al., 2020a) by *in vitro* culturing and subsequently appreciable amounts of secondary metabolites. It has been seen that among marine microorganisms, endophytic fungi of macroalgae show boundless potential to produce structurally diverse novel bioactive natural products (Zhang et al., 2016; Baracaldo et al., 2019; Kaddes et al., 2019). Most of these marine algal–derived fungi grow in an exceptionally extreme and inimitable habitat, which makes them capable to produce unusual secondary metabolites. This is simply because of their existence and adaptation to a distinct ecosystem (Flewelling et al., 2015; Wahaibi et al., 2019). Several studies have previously demonstrated the cytotoxic activities of marine endophytic extracts in cancer cells (Kamat et al., 2020a; Kamat et al., 2020b; Sajna et al., 2020). It has been observed that natural products provide apoptosis-modulating templates which can be extremely beneficial in management and therapy of cancer. Hence, it is imperative that apoptotic inducers, either in the form of crude extracts or as isolated compounds, be screened thoroughly (Motadi et al., 2020). In this study, we intended to evaluate the anticancer natural product potential of marine algae–associated endophytes. This could further help in developing novel lead molecules for developing drugs against cancer. To achieve this aim, four fungal strains associated with a marine alga *Sargassum muticum* were cultivated *in vitro* and tested for cytotoxic and induction of apoptosis by the fungal extracts.

*Sargassum muticum* is one of the common marine algae used in food and medicine in tropical and subtropical countries, including India. In traditional medicines, they are used as anticancer, antiviral, antidiabetic, anti-allergic, and antithrombotic agents (Zhu et al., 2006; Liu et al., 2012; Namvar et al., 2013).

Endophytic fungi residing inside *S. muticum* may also possess the bioactive potential as a result of genetic exchange and long-term symbiotic evolution with their host. Earlier, endophytic fungi and bacteria of plants used in traditional Chinese medicine have shown bioactive potential, very similar to their host plants (Miller et al., 2012).

Singh et al., 2016 have also studied the antioxidative activity of endophytic *Cladosporium velox*, and established its relationship with host plant *Tinospora cordifolia.*

**SCHEME 1:**
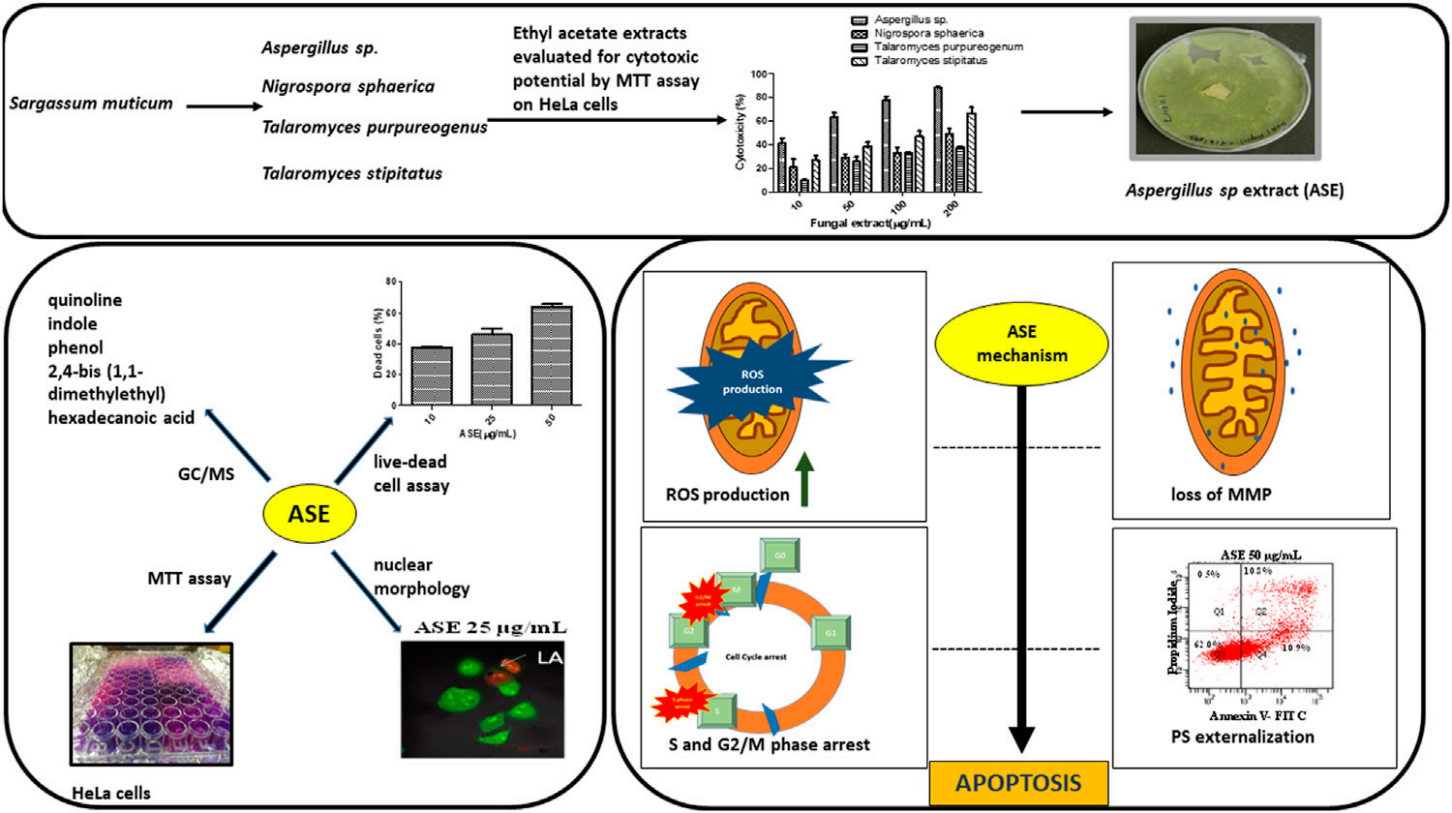
Schematic diagram representing the screening of isolated fungi, optimization of physicochemical parameters, their cytotoxic potential, and mechanism of cell death induced by ASE in HeLa cells.

Further, we report the cytotoxic activity and apoptosis induction by *Aspergillus* sp. ethyl acetate extract in HeLa cells. We support this observation by further investigating the molecular mechanisms triggering the event of apoptosis in HeLa cells (Scheme 1).

## Materials and Methods

### Materials

Fungal growth media, potato dextrose agar (PDA) and potato dextrose broth (PDB) were procured from Himedia, India. 3-(4, 5-dimethylthiazole-2yl)-2 and 5-diphenyl tetrazolium bromide (MTT) reagents were bought from SRL–Ranbaxy. Ethyl acetate, hexane, chloroform, and dichloromethane were procured from Merck, India. Dulbecco’s Modified Eagle’s Medium (DMEM), dimethyl sulfoxide (DMSO), 5,5′,6,6′-tetrachloro-1,1′,3,3′-tetraethylbenzimi-dazolylcarbocyanine iodide (JC-1), 2′,7′-dichlorodihydrofluorescein diacetate (DCFHDA), N-acetyl-L-cysteine (NAC), and Annexin-FITC/Propidium Iodide (PI) Staining Kit were procured from Sigma Aldrich, United States. Penicillin/streptomycin solution, GlutaMAX solution, Trypsin-EDTA, and fetal bovine serum (FBS) were bought from GIBCO-BRL. Doxorubicin was purchased from Calbiochem, India. Caspase 3, 7, and 10 Apoptosis Fluorometric Assay Kit was procured from G-Biosciences, United States. Apoptosis inhibitor “Z-VAD-FMK” was procured from Santa Cruz Biotechnology, United States. All chemicals and reagents used were of analytical grade.

### Human Cancer Cell Lines and Their Maintenance

HeLa (cervical adenocarcinoma), MCF-7 (breast adenocarcinoma), Hep G2 (hepatocellular carcinoma), A549 (lung carcinoma), A-431 (epidermoid carcinoma), and LN-229 (glioblastoma) and HEK 293T (human embryonic kidney cell line) were procured from National Center for Cell Sciences (NCCS) Pune, India. Dulbecco’s Modified Eagle’s medium (DMEM) supplemented with 10% FBS, 1% (v/v) penicillin/streptomycin solution, 1% (v/v) GlutaMAX solution was used to grow and maintain cell lines (HeLa, A-431, A549, LN-229, and HEK 293T) at 37°C in a humidified 5% CO_2_ atmosphere. Chinese Hamster Ovary (CHO), a non-cancer cell line was a kind gift from Prof. P. N. Rangarajan, Department of Biochemistry IISc Bangalore, India. Minimum Essential Medium Eagle (MEM) supplemented with 10% FBS, 1% (v/v) penicillin/streptomycin solution, 1% (v/v) MEM nonessential amino acid solution, and 1% (v/v) GlutaMAX solution was used to grow and maintain CHO cell line at 37°C in a humidified 5% CO_2_ atmosphere

### Isolation and Identification of Endophytic Fungi From a Seaweed

The seaweed *S. muticum* was collected from the Kovalam Coast, Thiruvananthapuram, Kerala, India, and brought to the laboratory along with sea water in a sterile polythene bag. Endophytic fungi were isolated adapting the protocol from Kjer et al., 2011. Further, the endophytic fungi were grown in PDB for 7 days at 28 ± 2°C, after which, genomic DNA was extracted. The internal transcribed spacer (ITS) region of rDNA was amplified and sequenced using primers ITS1 and ITS4. The sequences were compared with those available in GenBank using BLASTN. The isolated endophytic fungi were identified as *Aspergillus* sp., *Talaromyces purpureogenus*, *Nigrospora sphaerica*, and *Talaromyces stipitatus*. The ITS DNA sequences, thus obtained and downloaded sequences of their nearest neighbours were used to construct a phylogenetic tree using MEGA X (Minarni Artika et al., 2017; Kumar et al., 2018).

### Preparation of Endophytic Fungal Extracts

The four isolated endophytic fungi were pregrown on PDA for 10 days at 28 ± 2°C. Thereafter, two 5 mm diameter agar discs of each endophyte were used to inoculate 300 ml of PDB medium in a 1 L Erlenmeyer flask. These cultures were grown for 28 days at 28 ± 2°C in a 1 L flask in the dark and static conditions. After 28 days of incubation time, the fungal mycelia were separated from the supernatant and grinded in liquid N_2_. Supernatant and grinded mycelia were combined and extracted with twice the volume of ethyl acetate. The extracted organic phase was concentrated using a rotary evaporator (IKA RV 10 D S96) at 40°C. The residues were carefully weighed and solubilized in methanol (1 ml) and stored at 4 °C until further use.

### Assessment of *In Vitro* Cytotoxic Activity of Fungal Extracts by MTT Assay

The cytotoxicity of the extracts was examined by the MTT assay by determining the viability of cells (Kuriakose et al., 2014). Briefly, HeLa cells were grown and maintained in DMEM medium and supplemented with 10% fetal bovine serum, 100 U/ml penicillin, 100 mg/ml streptomycin, and 1% (v/v) GlutaMAX and incubated at 37°C in a 5% CO_2_ of the humidified chamber. 1 × 10^4^ cells/ml HeLa cells in 100 µL of DMEM were seeded in 96-well plates and incubated for 24 h. Thereafter, the cells were treated with fungal crude extracts at a range of concentrations (10, 50,100, and 200 μg/ml) in 100 µL media and incubated for 48 h, respectively. At the end of 48 h, 10 µL of MTT reagent (5 mg/ml) was added to each well. After 2 h of incubation at 37°C, the plates were emptied and MTT reagent was removed. To the each well, dimethyl sulfoxide (100 µL) was added and then the absorbance was measured at 595 nm wavelength with an ELISA reader Infinite M2000 Pro™ (Tecan, Crailsheim, Germany) (Kumari et al., 2018). The IC_50_ values were calculated for each fungal extract. All the assays were performed in triplicates and results were presented as mean ± SD. *Aspergillus* sp. extract demonstrated the highest cytotoxicity and was, hence, chosen for further investigation.

### Optimization of Incubation Interval, Extraction Solvent, and Growth Medium for Extraction of Cytotoxic Secondary Metabolites From *Aspergillus* sp.

In the first study, the growth curve of *Aspergillus* sp. was plotted. The fungus was inoculated in 300 ml of PDB and harvested after different time points (7, 14, 21, 28, and 35 days), and extracted with ethyl acetate. After optimization of incubation time, the organic solvent was optimized for increased extraction of anticancer secondary metabolites. In the second study, the endophytic fungus grown in PDB for 21 days was extracted with equal volume of different solvents of varying polarities viz. hexane, chloroform, dichloromethane, and ethyl acetate. . In the third study, nine liquid media were investigated for finding the suitable medium for growth and production of cytotoxic secondary metabolites viz. czapek yeast extract broth (CZB), malt extract broth (MEB), yeast extract phosphate broth (YEP), yeast malt extract broth (YME), potato dextrose broth (PDB), Goose and Tschessch broth (GTB), Leonine broth (LEB), Sabouraud broth (SDB), and Gauce medium (GA1) (Pu et al., 2013; Pan et al., 2016; Kumari et al., 2018) (Supplementary Table S1).

Each culture medium was inoculated with two agar discs (5 mm diameter) containing fungal mycelium, and incubated for 21 days at 28 °C under static conditions. The liquid cultures were carried out in 1 L Erlenmeyer flasks containing 100 ml medium. Each culture was grown in triplicate. At the end of the incubation period, the mycelial weight was recorded by filtering the contents of each culture flask through a preweighed Whatman no. 3 filter paper that was preweighed using Buchner funnel. The fungal mycelium was washed thrice with sterile deionized water. The fungal mycelium dry weight was measured after overnight drying in a hot air oven. As described earlier, the total culture fungal extract was prepared and investigated for cytotoxicity by the MTT assay.

### Analysis of Volatile Metabolites by Gas Chromatography–Mass Spectroscopy

The cytotoxic *Aspergillus* sp. ethyl acetate extract (ASE), was subjected to GC-MS analysis to investigate the presence of volatile metabolites. Previous reports have suggested that, GC-MS was used to analyze secondary metabolites from different organisms/sources, including bacteria (Mohamad et al., 2018), fungi (Gomathi et al., 2015; Kumari et al., 2018), and plants (Gomathi et al., 2015). High Performance Liquid Chromatography (HPLC) will again serve the same purpose. Many ethnopharmocology studies have also applied other spectroscopic techniques than HPLC to assess the chemical constituents of an extract (Ghuman et al., 2016; Mai et al., 2016; Savage et al., 2019).

The analysis of GC-MS was done in an Agilent GC-MS apparatus (GC: 7890A; MSD5975C) with fused silica HP-5 capillary column (30–0.25 mm, ID, thickness of film is 0.25 μm), was directly coupled to a single quadrupole MS. The Kavitha and Savithri (2017) protocol for chromatographic separation of the metabolites was followed. All the detected compounds were identified by mass spectral database search using the National Institute of Standards and Technology (NIST).

### Determination of Cytotoxic Activity of ASE on Different Cancer Cell Lines by MTT Assay

Cytotoxicity induced by ASE on a variety of human cancer cell lines viz. HeLa (cervical adenocarcinoma), MCF-7 (breast adenocarcinoma), Hep G2 (hepatocellular carcinoma), A549 (lung carcinoma), A-431 (epidermoid carcinoma) and LN-229 (glioblastoma), and on non-cancer human embryonic kidney cell line (HEK 293T) was determined by the MTT assay after 48 h of treatment, as described earlier (Kuriakose et al., 2014). HeLa cells treated with 1 mM doxorubicin for 16 h served as a positive control.

### Propidium Iodide Staining and Cell Viability Analysis by Flow Cytometry

To identify the percentage of viable cells upon treatment with ASE, a membrane impermeable dye that is generally excluded from viable cells, propidium iodide was used (Kuriakose et al., 2016). Cells stained with propidium iodide were counted as dead cells and unstained cells were counted as live/viable cells.

Briefly, the cancer cell line HeLa and a non-cancer cell line, CHO cells (1 × 10^4^ cells/ml) were seeded in a 24-well culture plate and treated with a range of concentrations of ASE (10, 25, 50 μg/ml) for 48 h. After 48 h, the cells were trypsinized, followed by PBS washes were given twice and PI (1 μg/ml) was added prior to data acquisition. Untreated healthy cells served as negative control, doxorubicin (1 mM) treated cells for 16 h served as a positive control. BD FACSVerse (Becton Dickinson, United States) was used for data acquisition and BD FACSDiva™ software was used to analyze the data.

### Quantification of Apoptosis Using Acridine Orange/Propidium Iodide Double Staining

The quantification of ASE-induced apoptotic HeLa cells was done by using acridine orange (AO) and propidium Iodide (PI) double staining. Each well of the six-well plate was seeded with 10^4^ HeLa cells approximately, on poly-L-Lysine (0.01%)–coated coverslips (24 mm). After 24 h of incubation, cells were subjected to 10, 25, and 50 μg/ml concentrations of ASE for 48 h. After 48 h, PBS is used for washing cells twice and then kept for staining for 2–3 min with a mixture of AO/PI (1 mg/ml). Under a confocal microscope (Epi-fluorescence Olympus DSU, Japan), these cells were surveyed for the presence of cell death by apoptosis (Zahedifard et al., 2015). Cells without treatment served as control.

### Determination of Mitochondrial Membrane Potential and Reactive Oxygen Species

The change in mitochondrial membrane potential (MMP or ΔΨm) after ASE treatment was determined by JC-1 staining as illustrated previously (Dhayanithy et al., 2019). As described earlier, HeLa cells (1 × 10^4^ cells) were seeded in 24-well plates and incubated for 24 h. On the following day, different concentrations on ASE were added to the medium. After 24 h of the addition of fungal extract, JC-1 dye (2.5 μg/ml) was added to each well and incubated for 15 min at 37 C in the CO_2_ incubator in dark conditions. Subsequently, the cells were trypsinized and washed with PBS. This was followed by an immediate analysis by flow cytometry at an excitation wavelength of 488 nm and emission wavelengths 530 nm and 590 nm. BD FACSVerse (Becton Dickinson, United States) was used for data acquisition and BD FACSSuite software was used to analyze the data. JC-1 monomers showed the emission around 530 nm (grezen fluorescence), while that of J aggregates exhibit at 590 nm (orange-red fluorescence). Cells treated with 2, 4-Dinitrophenol (2, 4-DNP) served as positive control, while the untreated cells served as a negative control.

ROS generation was measured in HeLa cells after treatment with ASE using DCFH-DA staining. Briefly, after 24 h of incubation of HeLa cells (1 × 10^4^ cells) in 24-well plates, the cells were treated with a range of ASE concentrations for 24 h. To confirm the production of ROS, ASE (50 μg/ml) was added with a ROS scavenger; N-acetyl-L-cysteine (NAC) at 5 mM concentration for 1 h (Badisa et al., 2015). In order to detect the production of ROS, HeLa cells after treatment were harvested, washed twice with PBS, and stained with 50 µM of DCFH-DA for a period of 15 min in dark at 37 C. Cell treated with H_2_O_2_ (800 µM) served as positive control and untreated cells served as negative control. The fluorescence generated as a result of oxidation after hydrolysis of DCFH-DA to DCFH was quantified using flow cytometry around 500 nm. BD FACSVerse (Becton Dickinson, United States) was used for data acquisition and BD FACSuite software was used to analyze the data.

### Measurement of Apoptosis by Phosphatidylserine Externalization by Annexin V-FITC/PI Staining

The exposure of phosphatidylserine (PS) is known to be a transitional occurrence through cellular apoptosis and stirring after mitochondrial membrane depolarization, nevertheless, before DNA fragmentation. Annexin V^+^/PI^−^ cells were quantified as early stage of apoptosis. However, Annexin V^+^/PI^+^ cells signify a late phase of apoptosis. The percentage of apoptotic HeLa cells treated with ASE was stained using the fluorescein isothiocyanate (FITC)–conjugated annexin V/Propidium Iodide (PI) Assay Kit, according to the manufacturer’s protocol (eBioscienceTM Annexin V; Invitrogen, San Diego, United States) and further quantified using flow cytometry. Briefly, HeLa cells (1 × 10^5^ cells) were seeded in 24-well plates in complete DMEM media and were allowed to adhere at 37 C in a CO_2_ incubator. After 24 h of incubation, the cells were treated with different concentrations (10, 25, and 50 μg/ml) ASE for 24 h. The cells were trypsinized, washed with PBS, and stained with 1 µL of Annexin V-FITC stain, with or without PI in an annexin-binding buffer, according to the manufacturer’s instructions. Cells were analyzed by flow cytometry after 15 min of incubation at room temperature in the dark at excitation/emission of 488/520 nm for Annexin-FITC and 540/630 nm for PI. BD FACSVerse (Becton Dickinson, United States) was used for data acquisition and BD FACSuite software was used to analyze the data.

### Cell Cycle Analysis

The effect of ASE on the cell cycle progression in HeLa cells was probed by adapting protocols from Zheng et al., 2016 with some modifications. HeLa cells (1 × 10^5^ cells) were seeded in 12-well plates in a complete DMEM media and allowed to adhere at 37 C in a CO_2_ incubator. After 24 h of incubation, the cells were treated with different concentrations of ASE for 24 h; harvested, washed twice with PBS, and 70% ethanol fixation was done, kept overnight at −20 C. After incubation, the fixed cells were centrifuged at 10,000 rpm for 10 min, washed twice with PBS, and then treated with RNase (5 μg/ml) for 4 h at 37°C. The cells were then stained with PI for 30 min prior to the acquisition of data by flow cytometry at 500 nm. BD FACSVerse (Becton Dickinson, United States of America) was used for data acquisition and BD FACSuite software was used to analyze the data. For each sample, 10,000 events were acquired. Untreated cells served as control.

### Caspase 3/7/10 Fluorometric Analysis

Briefly, 1 × 10^6^ HeLa cells were seeded in six-well plate. ASE was added with the increasing concentrations (25, 50, and 100 μg/ml), whereas untreated cells served as uninduced control. After 48 h of treatment, cells were centrifuged at 2,000 rpm, washed with PBS, and the cell pellet was stored at −20°C.

The Caspase 3, 7, and 10 Apoptosis fluorometric Assay Kit (G-Biosciences) was used to estimate the apoptotic activity of ASE on HeLa cells. Manufacturer’s instructions were followed to perform the assay. Briefly, the cell pellet was resuspended in 100 µL of lysis buffer and freeze/thawed for 4 to 5 times to obtain cell lysate. For each fluorometric reaction, 50 µL of cell lysate, 50 µL of 2X assay buffer containing 1 mM of DTT and 5 µL of 1 mM substrate “Aspartic acid–Glutamic acid–Valine–Aspartic acid–7-amino–4-trifluoromethyl coumarin” (DEVD-AFC) was added.

Caspases cleaved DEVD-AFC to release the peptide (DEVD) and fluorescent molecule (AFC), which can be read at emission 375 nm and excitation 530 nm. Untreated cells served as uninduced control containing 100 µL of assay buffer and 5 µL substrate AFC-DEVD. Two hundred µM of Z-VAD-FMK (Broad spectrum apoptosis inhibitor) added to 100 μg/ml ASE-treated cell lysate served as a negative control.

The reaction was carried out in 96-well plate. After taking readings at “*t* = 0,” reaction was incubated at 37°C for 60 min. The change in fluorescence absorbance at time point “0” minutes and time point “60” minutes was plotted in the graph.

### Statistical Analysis

All the experiments were performed in triplicate and quantitative variables were represented in terms of mean ± S.D. in histograms. For statistical significance, mean ± SD of all groups were compared and analysis of variance (ANOVA) was performed using a statistical package, SPSS 16.0 (SPSS Inc, Chicago, IL, United States). The probability of a *p*-value of ≤0.05 was taken to indicate statistical significance. Further, the Duncan’s multiple range test (DMRT) was used to identify the pairs of groups where the mean were significantly different at *α* = 0.05.

## Results and Discussion

### Four Endophytic Fungi Were Isolated and Identified From a Marine Alga, S. *muticum*

The fungal isolates were identified using a combination of classical and molecular taxonomy approach. In classical taxonomy approach, the fungi were identified based on their culture morphology. Four fungal endophytes recovered from *S. muticum* exhibited characteristic colony morphology on PDA (Table 1). *Aspergillus* sp. was characterized by the greenish-white growth of mycelia, while the reverse side was yellowish-orange to light peach in color. *N. sphaerica* demonstrated characteristics rapidly growing floccose white colonies, which turned black with age (Wang et al., 2017). *T. purpureogenus* was characterized by gray velutinous colonies, while *T. stipitatus* was identified by irregular white colonies, which turned gray with age. DNA fragments of around 500–600 bp (ITS region) were amplified and sequenced for all four isolates. The ITS sequences were compared with the GenBank sequences using a BLAST search and deposited in GenBank database (Table 1). Molecular characterization of these endophytes were done based on ITS rDNA sequence analysis. The isolated strains were morphologically and taxonomically identified as *Aspergillus* sp., *T. purpureogenus*, *N. sphaerica*, and *T. stipitatus*. The partial identified sequences have been submitted to GenBank with accession numbers MG807064, MG807063, MF457920, and MF457921, respectively (Table 1).

**TABLE 1:** Identification of endophytic fungi isolated from brown alga “*Sargassum muticum*.”

Strain number (endophyte)	Colony morphology^a^	Fungal strains identified^b^	GenBank accession number
1	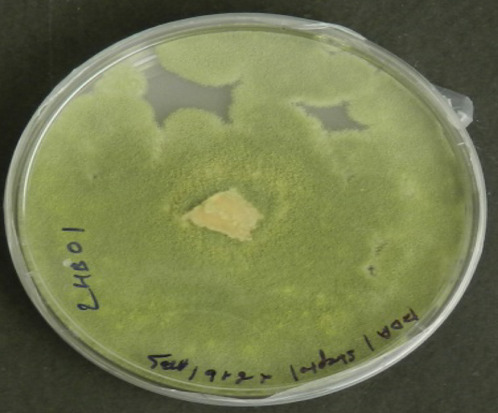	*Aspergillus* sp.	MG807064
2	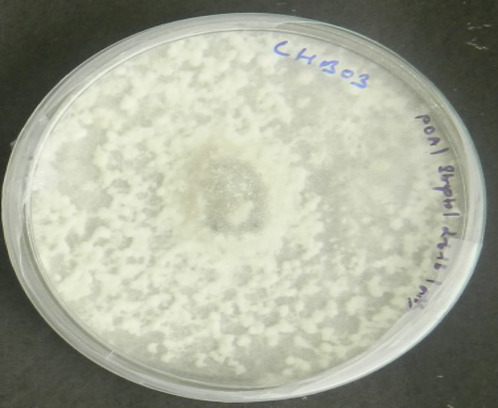	*Nigrospora sphaerica*	MF457920
3	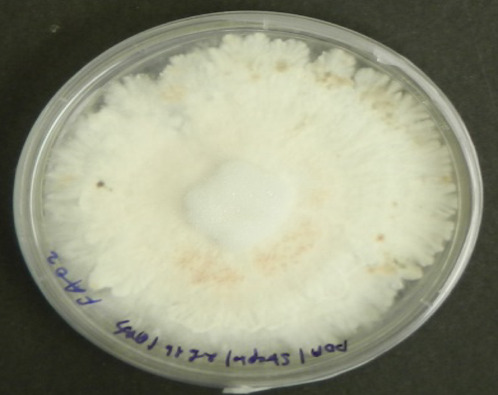	*Talaromyces purpureogenum*	MG807063
4	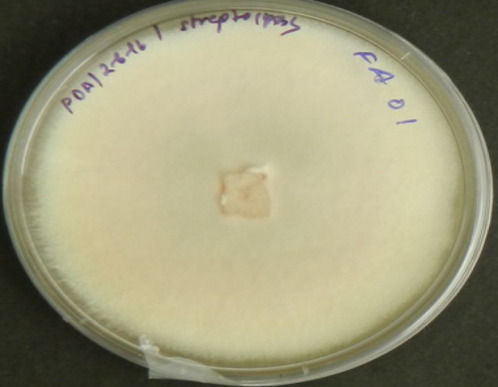	*Talaromyces stipitatus*	MF457921
			
			
			
			
			
			

a Colony morphology on PDA at 7 days.

b Molecular identification of fungal strains based on ITS rDNA sequence analysis.

**FIGURE 1:**
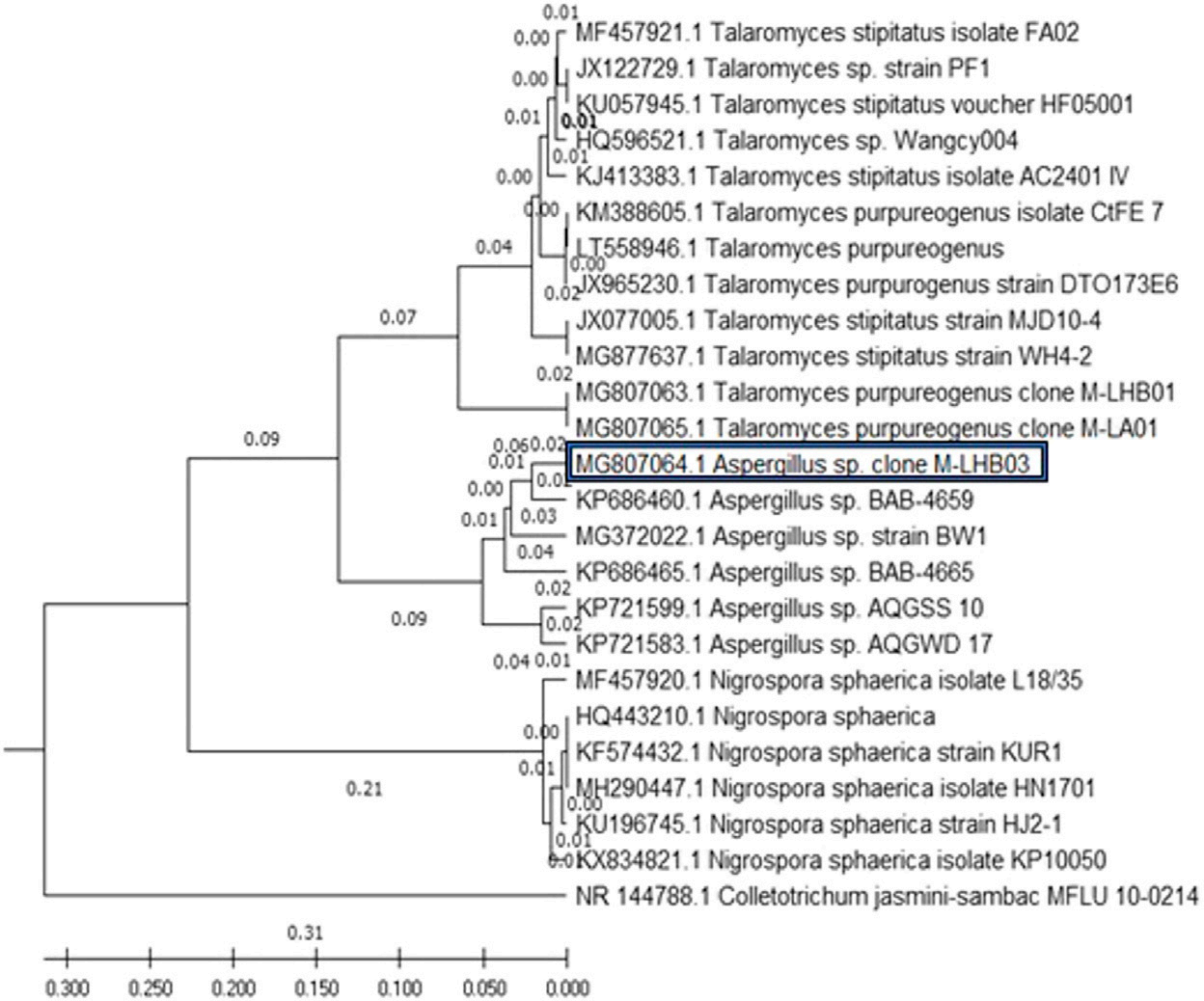
Phylogenetic relationship of four endophytic fungal isolates derived from *S. muticum* marine alga, sampled from the Kovalam Coast, was constructed using MEGA X software by the UPGMA method. The endophytic fungi clade with fungi of their respective genus and stand separate from the outgroup *Colletotrichum jasmine-sambac.*

The evolutionary history of the five endophytic fungi was constructed using the MEGA X software and deduced using the UPGMA method. Figure 1 shows the optimal tree with the sum of branch length = 1.30066823. The tree was drawn to scale, with branch lengths, as represented next to the branches, in the same units as those of the evolutionary distances used to deduce the phylogenetic tree. The maximum composite likelihood method was used to compute the evolutionary distances in the tree. It was observed that all the four endophytic fungi clade with endophytes of their respective genus and stand separate from the outgroup *Colletotrichum jasmine-sambac* (Katoch et al., 2017; Minarni Artika et al., 2017; Kumar et al., 2018).

### The Crude Extracts of Isolated Endophytic Fungi Demonstrated Cytotoxic Activity on Cancer Cells

**FIGURE 2:**
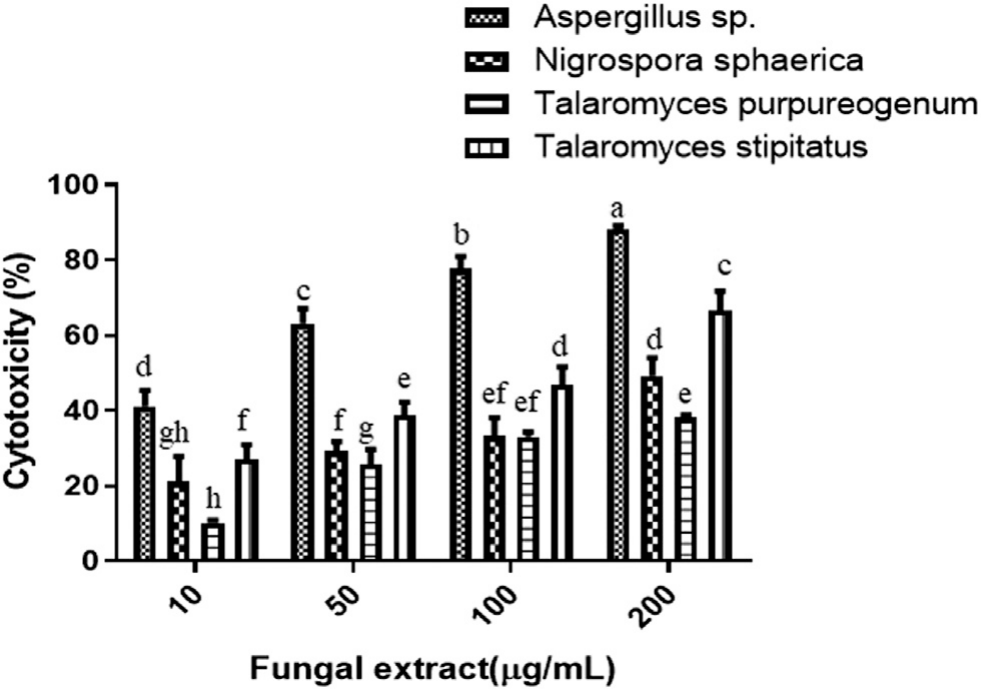
Four endophytic fungal crude extracts were tested for cytotoxicity on the HeLa cells. Different concentrations of fungal crude extracts were used to treat cells for 48 h, and cytotoxicity was determined by the MTT assay. Means sharing different letters differ significantly from each other at *p* ≤ 0.05.

The four fungi were grown for 35 days in PDB to test for their cytotoxicity, and extraction of fungal mycelium together with the culture filtrate was done with ethyl acetate. The cytotoxicity of the dried fungal extract was investigated on HeLa cells. There was a dose-dependent response when four different concentrations (10, 50, 100, and 200 µg/ml) were tested for each sample. The results of the cytotoxic activity of the fungal crude extracts are shown in Figure 2. The IC_50_ values of *Aspergillus* sp. and *T. stipitatus* extracts for HeLa cell were 30 ± 1 and 117 ± 2 µg/ml, respectively, while for *T. purpureogenus* and *N. sphaerica* extracts, the IC_50_ values were ≥200 µg/ml for HeLa cells. Among the four endophytic isolates screened, *Aspergillus* sp. demonstrated maximum cytotoxic activity on HeLa cell line after 48 h of treatment in a concentration-dependent manner, and hence chosen for further studies (Figure 2). Endophytic fungi residing in pharmacologically important algae may also gain specific attributes or contribute to their host (Gomes et al., 2015; Mayer et al., 2020) to adapt the marine niche, resulting in the production of bioactive compounds.

*Penicillium* and *Aspergillus* sp. have earlier been reported as endophytes of the marine alga *Sargassum* (Flewelling et al., 2015), but their role as cytotoxic agents have not been elucidated. Earlier, Miao et al., 2014 reported anticancer activity of endophytic *Aspergillus wenti* isolated from *Sargassum fusiforme.*

## Growth Parameters Optimization Resulted in Increase in the Production of Cytotoxic Secondary Metabolites From Total Culture Extract of *Aspergillus* sp.

### Growth Curve Optimization

**FIGURE 3:**
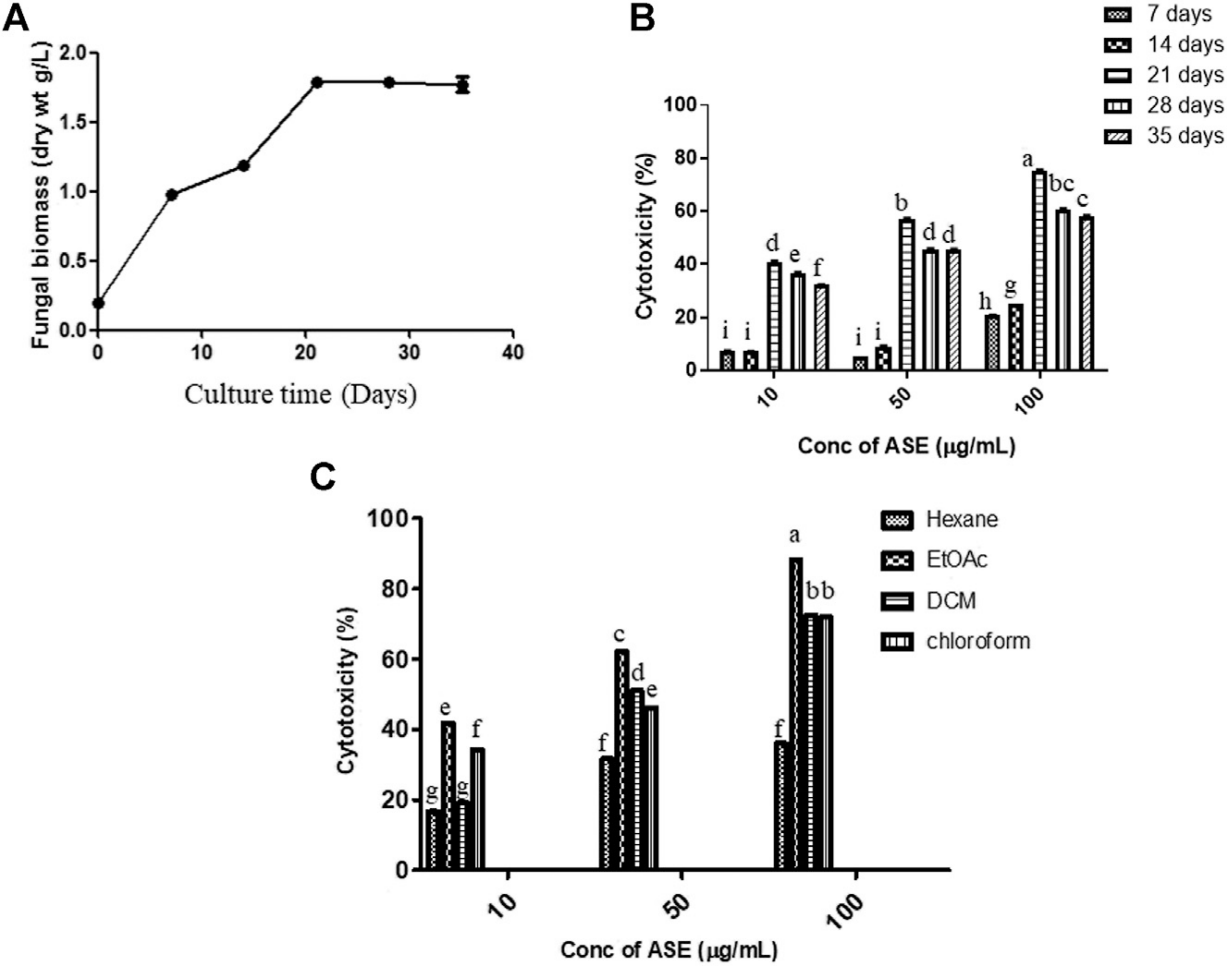
Time course of production of **(A)** biomass, **(B)** cytotoxic secondary metabolites in PDB culture of *Aspergillus* sp., and **(C)** effect of the different solvent extract on cytotoxicity. The fungus was grown for the different time intervals in PDB. The mycelium was harvested, dried, and weighed as described in Materials and Methods. cytotoxic effect of the extracts on HeLa cells was determined by the MTT assay. Means sharing different letters differ significantly from each other at *p* ≤ 0.05.

Fungal isolate was grown in PDB for different time intervals of 7, 14, 21, 28, and 35 days under stationary condition. Maximum dry biomass (1.7 g/L) and cytotoxic activity were obtained after 21 days of inoculation in total culture ethyl acetate fungal extract (Figures 3A,B). Bioactive compounds present in the fungal extract are generally secondary metabolites which were produced in stationary phase of growth curve, which was reached after 21 days of incubation with this extract (Bourgaud et al., 2001; Kebede et al., 2017; Navarri et al., 2017). The results well corroborated with the highest cytotoxicity obtained after 21 days of incubation (Figure 3B), which was also the time period for highest production of secondary metabolites. Further, since production was carried out in batch process, there is also nutrient depletion, with Carbon and N-source limitation, inhibiting the increase in cytotoxic activity after 21 days of incubation.

### Solvent Optimization

After optimization of incubation time, different solvent systems viz. hexane, ethyl acetate, dichloromethane, and chloroform were used for extracting cytotoxic secondary metabolites from the total culture fungal extract grown in PDB for 21 days (Figure 3C). Maximum cytotoxicity was obtained in ethyl acetate extract of total culture fungal extract, followed by dichloromethane. After optimization of different growth parameters, the IC_50_ value obtained was 21.35 ± 0.9 μg/ml against HeLa cell line. The polarity of metabolite is a characteristic that must be considered during extraction optimization (Haque et al., 2007). Maximum activity in ethyl acetate extract demonstrates semipolar nature of bioactive compounds responsible for cytotoxic activity.

### Media Optimization

**FIGURE 4:**
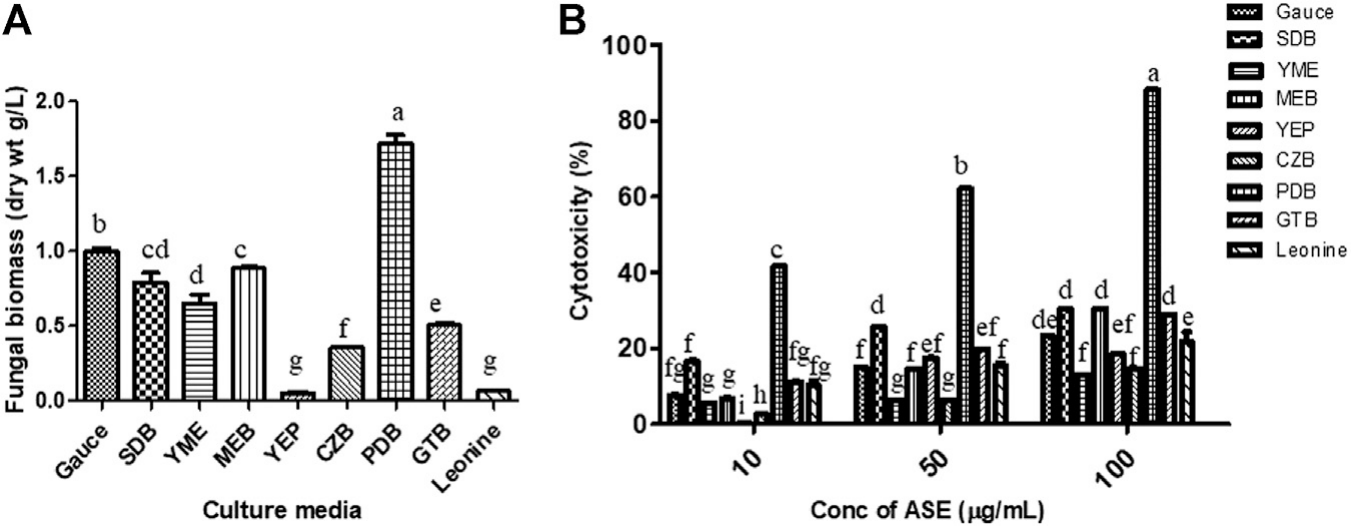
**(A)** Biomass. **(B)** *Aspergillus* sp. was grown in different media for the production of cytotoxic secondary metabolites. *Aspergillus* sp. grew in different culture media; the mycelium was separated, dried, and weighed as described in Materials and Methods. Cytotoxicity of ASE was tested on HeLa cells by the MTT assay. Means sharing different letters differ significantly from each other at *p* ≤ 0.05.

To find the optimum culture medium for the production of maximum biomass and the cytotoxic secondary metabolites, *Aspergillus* sp. was grown in nine different growth media, namely, czapek yeast extract broth (CZB), malt extract broth (MEB), yeast extract phosphate broth (YEP), yeast malt extract broth (YME), potato dextrose broth (PDB), Goose and tschessch broth (GTB), Leonine broth (LEB), sabouraud broth (SDB), and Gauce medium (GA1). *Aspergillus sp.* was grown for 21 days in abovementioned media, and the total culture was extracted with ethyl acetate, which was further evaluated for their cytotoxic activity. Among the nine media used, maximum biomass was obtained in PDB (1.7 g/L), followed by Gauce medium, MEB, and SDB (Figure 4A). Maximum cytotoxicity was observed in ASE obtained from PDB after 21 days of inoculation, followed by MEB, SDB, and CZB (Figure 4B). Media are a complex source of all the nutritional requirements of fungus and its manipulation will result in higher or lower production of cytotoxic compounds (Xu, 2015; Al-ansari et al., 2020). The different combination of carbon, nitrogen, growth supplements, and pH can significantly alter the production of desired secondary metabolites responsible for the cytotoxic activity. Understanding and exploiting the endophytes of ethnopharmacologically important algae, especially marine algae which have been used since ages may allow the discovery and sustainable production of the desired novel product.

The results demonstrated that all the different extracts of *Aspergills* sp. in different parameters (time, solvent and media) exhibited some cytotoxicity against HeLa cells, but ethyl acetate extract grown in PDB showed significantly higher cytotoxicity; hence, this extract (ASE) was used for further work.

### ASE Demonstrated Cytotoxicity on Multiple Human Cancer Cell Lines

**FIGURE 5:**
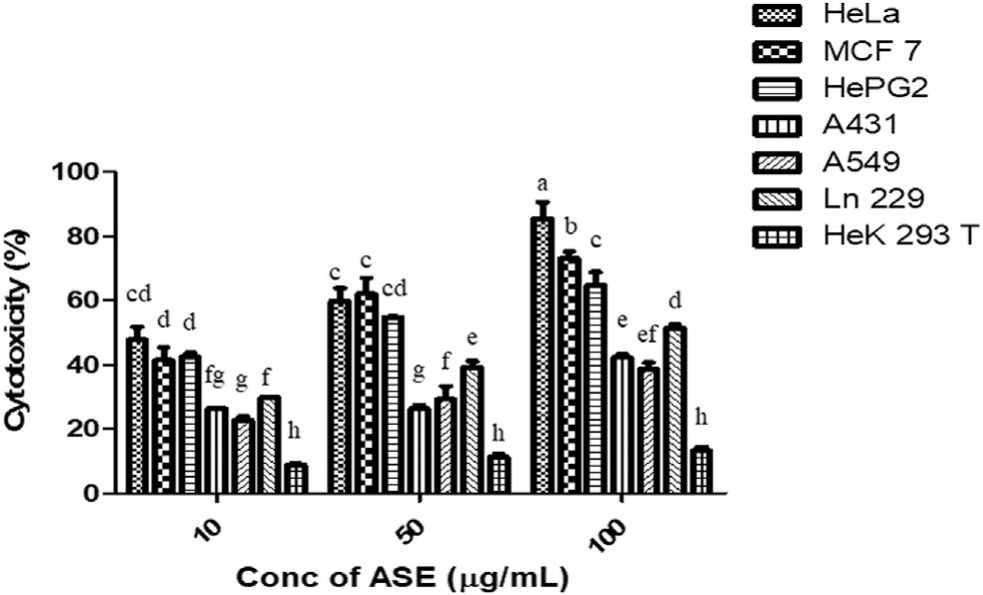
Cytotoxicity of ASE on multiple human cancer cell lines. Different concentrations of ASE were used to treat cells for 48 h, and cytotoxicity was examined by the MTT assay. Means sharing different letters differ significantly from each other at *p* ≤ 0.05.

To test the broad-spectrum cytotoxicity of ASE, it was evaluated for cytotoxicity *in vitro* against six human cancer cell lines, HeLa (cervical adenocarcinoma), MCF-7 (breast adenocarcinoma), Hep G2 (hepatocellular carcinoma), A549 (lung carcinoma), A-431 (skin/*epidermis* carcinoma) and LN229 (glioblastoma), and HEK 293T (normal human embryonic kidney cell line) by the MTT assay. ASE exhibited a different degree of cytotoxicity toward these cell lines in a dose-dependent manner (Figure 5). HeLa cells showed maximum cytotoxicity with IC_50_ value of 24 ± 2 µg/ml, followed by MCF-7 and Hep G2 with IC_50_ values of 32.0 ± 2.3 µg/ml and 33.0 ± 2.3 µg/ml, respectively. The ASE showed moderate cytotoxicity on A549, A-431, and LN229 with an IC_50_ value of 166.6 ± 1.4, 155.0 ± 1.3, and 94.8 ± 2.2 µg/ml, respectively. Many natural products can target a range of cancer cell lines, making them a potential candidate for novel drug discovery or enhancing the potential of existing drugs (Shakeri et al., 2018; Spradlin et al., 2019).

The ASE did not show cytotoxicity on HEK 293T non-cancerous human embryonic kidney cell line cells, indicating that ASE is not toxic to non-cancerous cells. One of the most important criteria for testing the safety of cytotoxic drug is their cytotoxic effect on non-cancer cell lines. Efficient cytotoxic drugs implant different mechanisms on cancer or non-cancer cells, making cancerous cells more susceptible toward the drug (Navya et al., 2019). Krishnamurthy et al., 2018 characterized an ankaflavin from *Penicillium aculeatum* and found significantly less cytotoxicity on HEK 293T and CHO cell lines. HeLa cells were used for further studies.

### Secondary Metabolites Were Analyzed by Gas Chromatography–Mass Spectroscopy

**FIGURE 6:**
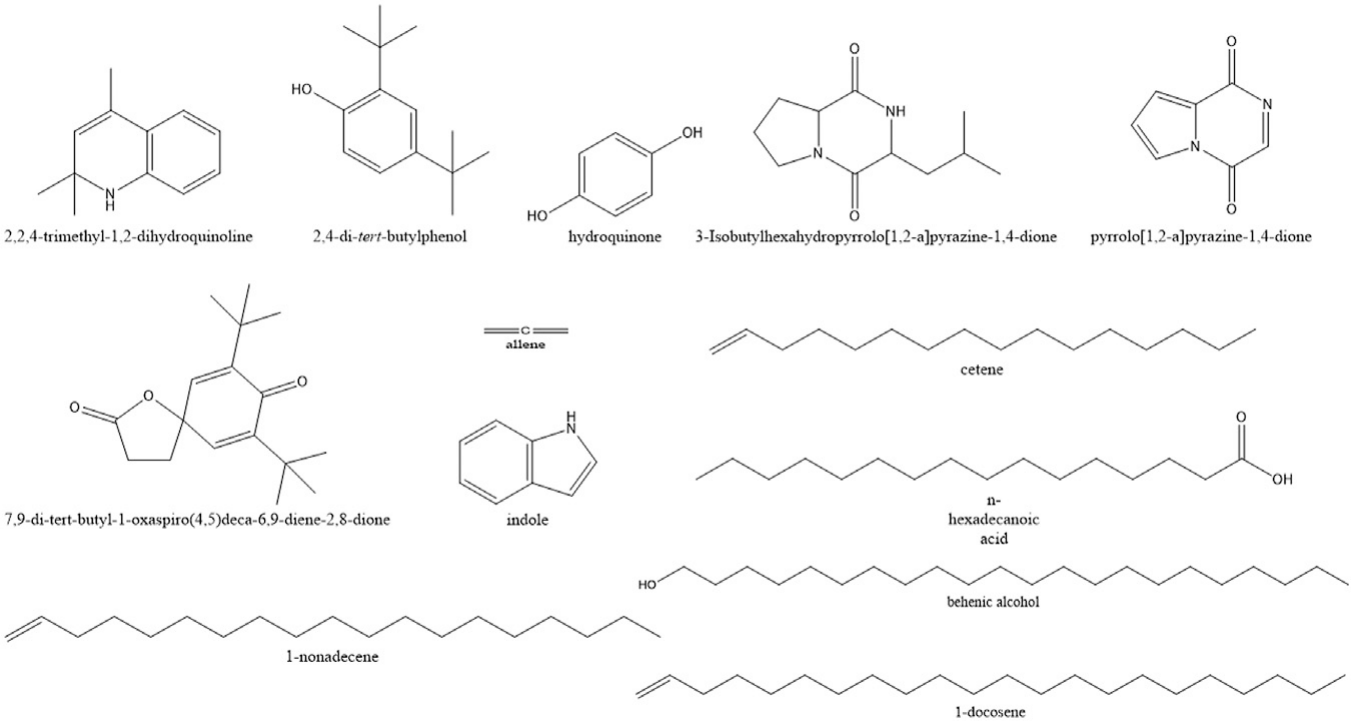
Structures of the major compounds detected in GC-MS analysis of ethyl acetate extract of *Aspergillus* sp.

A total of 71 compounds were identified after GC-MS analysis of the total culture fungal extract (Table 2). Many peaks were not identified using GC-MS database, suggesting the existence of novel compounds which can also be responsible for cumulative cytotoxicity of ASE. 1,2-dihydro-2,2,4-trimethylquinoline; 2,4-Bis(1,1-dimethyl ethyl)phenol; cetene; indole; hexahydro-3-(2-methyl propyl); 7,9-di-tert-butyl-1-oxaspiro(4,5)deca-6,9-diene-2,8-dione; pyrrolo[1,2-a]pyrazine-1,4-dione; hexahydro-3-(2-methyl propyl); behenic alcohol; 1-docosene; n-hexadecanoic acid; and 1-nonadecene were some of the major compounds (Figure 6).

**TABLE 2:** Quantification of metabolites present in the ethyl acetate total culture extract.

S. no	RT (min)	Metabolites	Relative concentration (%)
1	3.829	Isophorone	0.024739 ± 0.0214
2	4.39	Dehydromevalonic lactone	0.089144 ± 0.784
3	4.659	Benzenemethanol, α-methyl-, acetate (1-Phenylethyl acetate)	0.007911 ± 0.06
4	4.869	Allene	0.002334 ± 0.02
5	5.185	2,5-Diamino-6-methyl-4-pyrimidinol	0.041644 ± 0.06
6	5.268	Cyclopentanecarboxamide, N-[2-(2-methylphenoxy) ethyl]	0.0256 ± 0.01
7	6.027	Hydroquinone	0.038706 ± 0.003
8	7.61	1,2-Benzenedicarboxylic acid	0.029598 ± 0.214
9	8.411	Indole	0.118424 ± 0.002
10	8.411	4-Thiepanone, 5-hydroxy-3,3,6,6-tetramethyl	0.32368 ± 0.002
11	8.865	Benzaldehyde, 4-hydroxy	0.468704 ± 0.004
12	8.895	Nicotinonitrile, 2-mercapto-5,6-dimethyl	0.276363 ± 0.00.3
13	8.928	Propanoic acid, 3-chloro-, 4-formylphenyl ester	0.024365 ± 0.008
14	8.98	2-Hydroxyethyl salicylate	0.208081 ± 0.005
15	9.348	Phenol, 2-propyl	0.231031 ± 0.001
16	10.268	1-Tetradecene	0.215665 ± 0.004
17	10.245	Acetic acid, (2,4-xylyl)	0.105721 ± 0.06
18	12.494	4H-Pyran-4-one, 5-hydroxy-2-(hydroxymethyl)	0.057381 ± 0.05
19	13.215	Quinoline, 1,2-dihydro-2,2,4-trimethyl	0.46619 ± 0.01
20	13.241	Benzene, (1-methyl-2-cyclopropen-1-yl)	0.096929 ± 0.01
21	13.966	Pentanamide, 2-amino-4-methyl-, (S)	0.223893 ± 0.008
22	14.638	2,5-Cyclohexadiene-1,4-dione, 2,6-bis(1,1-dimethylethyl)	0.17996 ± 0.007
23	15.4757	2H-Pyran-2-one, 5,6-dihydro-6-pentyl	0.010647 ± 0.02
24	16.004	3-Furanacetic acid, 4-hexyl-2,5-dihydro-2,5-dioxo	0.493393 ± 0.008
25	16.075	Hexathiane	0.139576 ± 0.02
26	18.319	Phenol, 2,4-bis(1,1-dimethylethyl)	16.23937 ± 0.008
27	21.718	Pentaerythritol tetraacetate	0.205363 ± 0.005
28	25.404	4-Ethoxy-3-anisaldehyde	0.096244 ± 0.001
29	27.392	4-Trifluoroacetoxyhexadecane	0.006329 ± 0.004
30	27.449	Cetene	0.312184 ± 0.06
31	27.506	7-Tetradecene, (E)-	0.00283 ± 0.05
32	30.629	Benzenesulfonamide, 2-methyl	0.017773 ± 0.004
33	37.441	Benzenesulfonamide, 4-methyl	0.0041 ± 0.145
34	58.002	2-Dodecylcyclobutanone	0.293613 ± 0.002
35	57.694	1-(3,3,3-Trifluoro-2-hydroxypropyl) piperidine	0.072175 ± 0.004
36	76.726	1-Decanol, 2-hexyl	0.008129 ± 0.002
37	123.608	Benzothiazole, 2-(2-hydroxyethylthio)	0.151434 ± 0.03
38	125.498	Pyrrolo [1,2-a] pyrazine-1,4-dione, hexahydro-3-(2-methylpropyl)	2.502151 ± 0.01
39	125.269	7,9-Di-tert-butyl-1-oxaspiro (4,5)deca-6,9-diene-2,8-dione	2.681396 ± 0.03
40	125.315	Pyrrolo [1,2-a] pyrazine-1,4-dione, hexahydro-3-(2-methylpropyl)	1.132229 ± 0.04
41	127.521	Hexadecane, 1,1-bis(dodecyloxy)	0.01824 ± 0.0254
42	127.555	Dibutyl phthalate	0.265049 ± 0.005
43	128.671	Behenic alcohol	14.00159 ± 0.02
44	128.701	1-Docosene	7.517438 ± 0.03
45	129.326	2-Oxabicyclo [4.2.0]oct-4-en-3-one, rel-(1R,6S,7S,8S)-5-methoxy-8-(4-methoxy-2-oxo-2H-pyran-6-yl)-7-(4-ethylphenyl)-1-[(E)-2-(4-ethylphenyl)ethenyl]	2.23088 ± 0.008
46	129.331	n-Hexadecanoic acid	5.130184 ± 0.339
47	129.396	7-[2-(Ethoxycarbonyl)-3α,5β-dimethoxycyclopentyl-1]-heptanoic acid, ethyl ester	0.023503 ± 0.1
48	129.914	1-Nonadecene	15.90127 ± 0.004
49	129.834	Behenic alcohol	0.824719 ± 0.005
50	132.75	Fluoranthene	0.032659 ± 0.654
51	132.752	Pyrene	0.014283 ± 0.006
52	133.556	cis-10-Heptadecenoic acid	0.824719 ± 0.0215
53	134.549	3,7,11,15-Tetramethyl-2-hexadecen-1-ol	0.019086 ± 0.009
54	135.349	E-8-Octadecacen-1-ol acetate	0.149983 ± 0.006
55	136.106	10-Undecen-1-ol	0.00408 ± 0.001
56	136.667	Dodecane, 1-iodo	0.011406 ± 0.339
57	144.356	1-Docosene	0.165953 ± 0.19
58	138.27	2-Ethyl-1-dodecanol	0.023392 ± 0.02
59	139.533	1,11-Dodecadiene	0.010846 ± 0.001
60	142.191	2-Methyl-1-octadecene	0.378232 ± 0.002
61	142.866	Octadecanoic acid	0.339807 ± 0.02
62	145.377	Behenic alcohol	22.31756 ± 0.007
63	149.738	Tetradecane	0.007353 ± 0.01
64	149.784	4,4-Dipropylheptane	0.002693 ± 0.002
65	154.351	1-Heptene	0.003067 ± 0.02
66	161.002	Cyclotetracosane	0.043148 ± 0.08
67	161.161	n-Tetracosanol-1	2.719422 ± 0.08
68	169.467	2-Butyl-1-decene	0.014163 ± 0.01
69	173.972	2,3,4-Trimethyl-1-pentanol	0.004776 ± 0.002
70	179.549	Tetracosyl heptafluorobutyrate	0.005889 ± 0.08
71	179.654	1-Tricosanol	0.029586 ± 0.001

*Aspergillus sp*. by GC-MS analysis. Values are the mean ± SD of three independent experiments.

Quinoline and indole act as precursors for construction motif of many novel anticancer drugs (Wang et al., 2015; Jain et al., 2016). 2,4-bis(1,1-dimethylethyl) phenol, a metabolite produced by seaweed-associated bacteria, *Gracilaria gracilis*, was shown to inhibit quorum sensing by disrupting biofilm formation in the uropathogenic *Serratia marcescens* (Padmavathi et al., 2014). Hexadecanoic acid isolated from leaves of *Kigelia pinnata* showed the cytotoxic effect on HCT-116 (human colorectal cancer) by DNA topoisomerase I interaction (Ravi and Krishnan, 2017). Allene and hydroquinone were also produced from the fungal extract which has been used as antiviral and anticancer compounds against melanoma cells, respectively (Hoffmann-Röder and Krause, 2004; Byeon et al., 2018).

Many compounds produced by endophytic isolate *Aspergillus* sp. from *S. muticum* were found to have biological activities. Fungal endophytes isolated from ethnopharmacologically important plant and algae can become an inexpensive alternative to natural products.

## Treatment of ASE Resulted in Interference With Cell Viability, Progression in Cell Cycle, and Cell Nuclear Morphology in HeLa Cells

### PI Live/Dead Analysis Revealed the Concentration-Dependent Cytotoxicity of ASE in HeLa Cells

**FIGURE 7:**
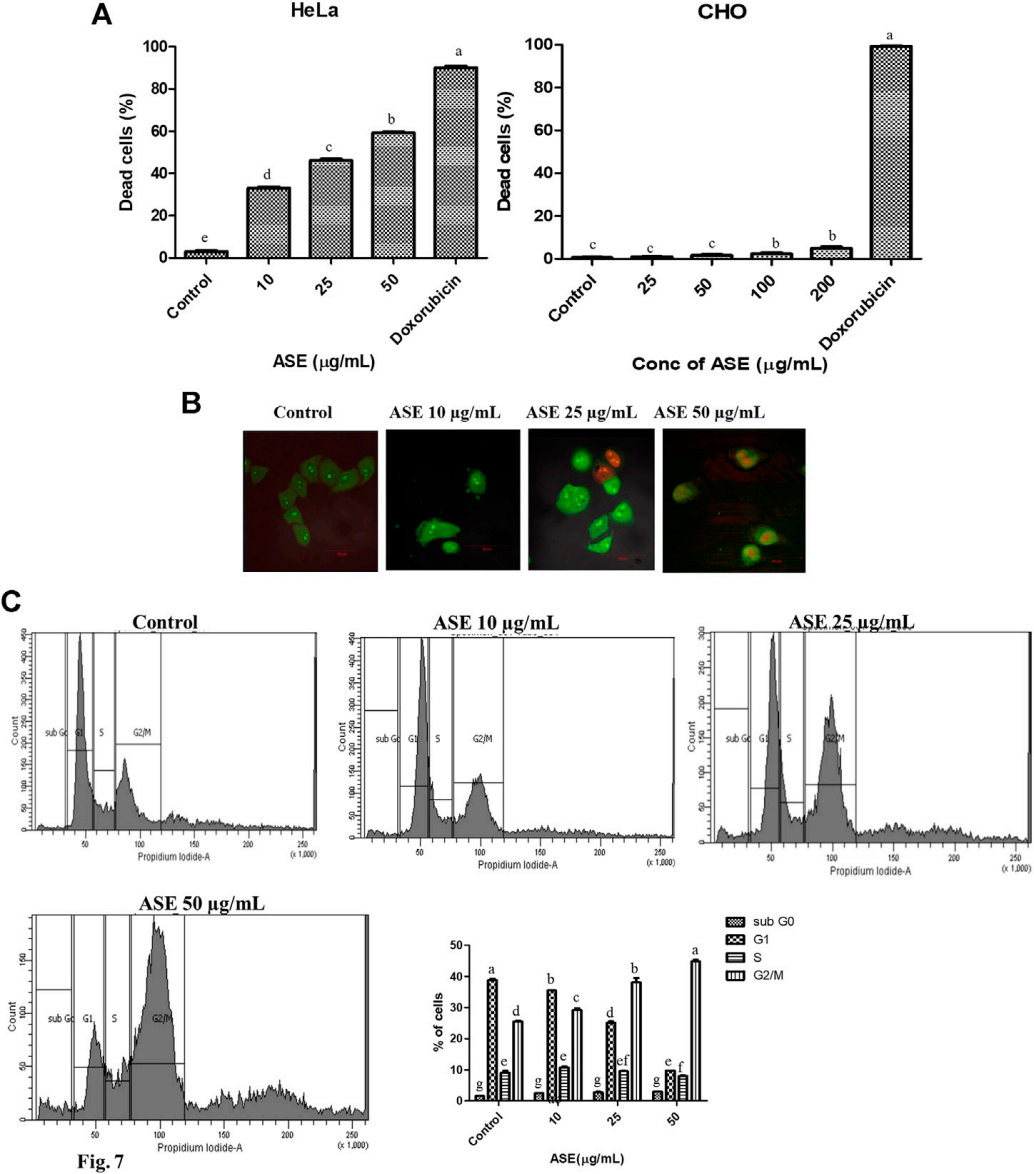
Effects of ASE on HeLa cell line including the viability of cells, cell cycle distribution, and cellular morphology. Treatment of cells with different concentrations of ASE **(A)** PI-based live/dead staining by flow cytometry analysis upon treatment with ASE. The percentage of dead cells was shown in the ASE-treated HeLa cells and non‐cancer CHO cells in a bar diagram. **(B)** Fluorescent micrographs of apoptotic morphological changes in HeLa cells treated with ASE followed by dual staining with AO/PI. Apoptotic/dead cells emit red fluorescence, while live/viable cells emit green fluorescence. The arrows indicate apoptotic cells. **(C)** The percentage of cells in each cell cycle phase was evaluated by propidium iodide–based staining by flow cytometry in the HeLa cells after treatment with ASE. The distribution of cells at different phases of cell cycle progression following treatment with ASE represented in a bar diagram. Means sharing different letters differ significantly from each other at *p* ≤ 0.05.

To evaluate the cytotoxic effect of ASE on HeLa cells, PI-based cell live/dead staining was employed. The PI live/dead assay is a reliable method to quantify the dead cells, where PI is able to stain nuclear material only when the membrane is compromised (Kumari et al., 2018; Kamat et al., 2020b). HeLa cells were treated with indicated concentrations of ASE (10, 25, and 50 μg/ml), stained with PI, and flow cytometry analysis was done (Supplementary Figure S1). The PI live/dead assay was also performed with a non-cancer CHO cell line. Figure 7A showed an increase in dead cell population with a dosage-dependent effect on viability after 48 h treatment on HeLa cells, while CHO cell line was not affected by increased concentration of the drug. The percentage dead cell population were found to be 38 ± 0.9, 50 ± 1.2, and 60 ± 1.3 μg/ml for HeLa cells, respectively, when treated with 10, 25, and 50 μg/ml of ASE. These observations confirm cytotoxicity of ASE in HeLa cells, while they prove to be non-cytotoxic on non-cancer CHO cell line. Doxorubicin showed a significant cytotoxicity on both HeLa and CHO cell lines. Natural products are known to be cytotoxic on cancer cell lines, while having no inhibitory action on normal cells on similar concentration. Artemisinin, a repurposed cytotoxic natural product derived from *Artemisia annua* L. shows potent cytotoxicity against retinoblastoma cell lines, while it shows low cytotoxicity on normal retina cell lines (Lichota and Gwozdzinski, 2018). The changes in the cell surface, microtubule remodeling, dysfunctional ROS scavenging system, cell cycle, and DNA repair mechanisms are some of the parameters which are significantly altered in cancer cells as compared to normal cells, leading to different mode of action of natural products and different cytotoxicity on the normal and cancer cells (Borys et al., 2020). Doxorubicin, on the other hand is a potent anticancer drug, but has been known for several side effects, including non-targeted cytotoxicity (Ibiyeye et al., 2019). The cytotoxic compounds isolated from the extract of ASE can help in reducing the side effects of chemotherapy.

### Change in Nuclear Morphology of HeLa Cells was Shown by AO/PI Staining

AO is a cell permeable fluorescent dye which stains nuclear DNA in both live and dead cells, whereas PI is another fluorescent dye that stains nuclear DNA in only those cells that have lost their membrane integrity (Astuti et al., 2016). AO/PI staining is a common morphology–based method to differentiate between normal, early apoptotic, and late apoptotic cells. In order to study nuclear morphological changes associated with in HeLa cells upon treatment with ASE, acridine orange and propidium iodide dyes were used for fluorescence microscopy. As shown in Figure 7B, untreated cells showed light green fluorescence while apoptotic cells showed bright green fluorescence, indicating chromatin condensation and membrane blebbing in ASE-treated cells. Propidium iodide binding and labeling to DNA, indicated by red color established that the late apoptosis stage affected by the ASE membrane blebbing is a key feature of early apoptosis which results from caspase-mediated activation of ROCK I (Coleman et al., 2001). Similarly, different stages of chromatin condensation can reveal the different stages of apoptosis (Tone et al., 2007).

### G2/M Phase Cell Cycle Arrest was Observed After Treatment With ASE in the HeLa Cells

To get an insight whether cytotoxicity induced by ASE, implicates changes in the progression of the cell cycle, cells in different stages of the cell cycle were analyzed by flow cytometry. As shown in Figure 7C, we observed a concentration-dependent increase in accumulation of cells in G2/M phase after 48 h of treatment in ASE-treated cells at 10, 25, and 50 µg/ml and there was a decrease in G1 and S phase cells. These results show ASE causes arrest at a G2/M phase in cell cycle progression. The connection with the apoptosis and cell cycle arises from the multiple indications that cell cycle progression alteration may either block or trigger apoptosis (Pucci et al., 2000). Natural products are known to induce G2/M phase arrest, thereby inducing apoptosis (Hnit et al., 2020; You et al., 2020). Astuti et al., (2016) observed apoptosis-mediated cell death by endophytic *Aspergillus* sp,, isolated from *Piper crocatum* after inducing S phase cell cycle arrest. Several reports support the involvement of apoptosis regulatory genes in cell cycle progression as well (Pucci et al., 2000; Wang et al., 2016).

## The Mechanism Involved in the Induction of Apoptosis by ASE in the HeLa Cells

### DCFH-DA Staining Detected Production of ROS After Treatment of ASE in HeLa Cells

**FIGURE 8:**
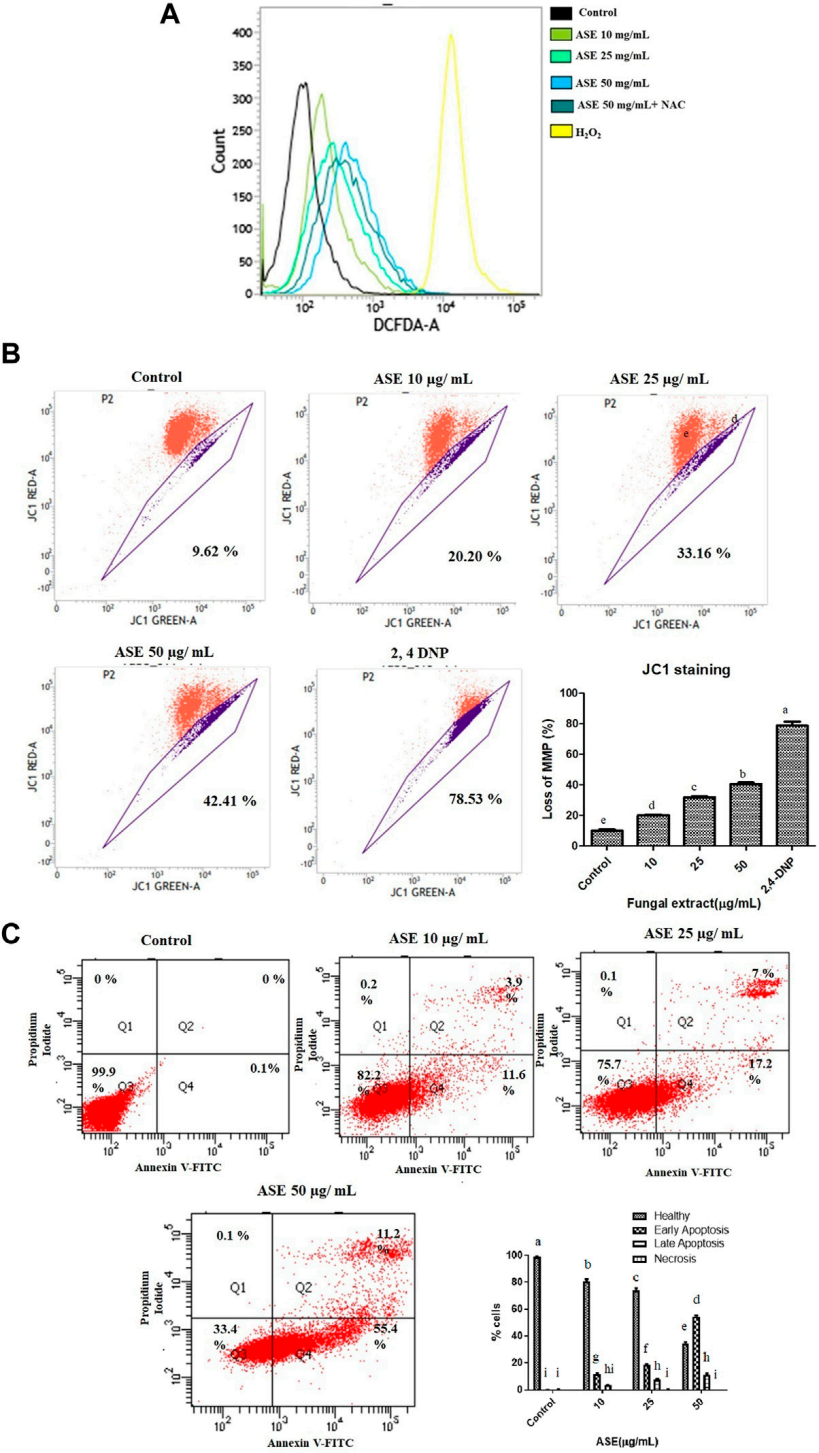
Effect of ASE on HeLa cell apoptosis. **(A)** Effect of ASE on ROS production. Intracellular ROS was detected by DCFH-DA dye after cells were treated with ASE by flow cytometry. Overlay graph presents as fold values using graphs compared with control. **(B)** Effect of ASE on mitochondrial membrane depolarization. Cells treatment was with different concentrations of ASE for 24 h and then they were stained with JC-1 which detects depolarization of mitochondrial membrane by using flow cytometry. The percentage of ASE-treated cells showing the increase in mitochondrial membrane depolarization in a dosage-dependent manner was shown in a bar diagram; cells treated with 2,4-dinitrophenol (2,4-DNP) served as positive control. **(C)** Apoptosis (PS externalization) determined by propidium iodide uptake and binding Annexin V to phosphatidylserine of by flow cytometry analysis. It shows four group of cells, cells that were negative for Annexin-V and propidium iodide were normal healthy (Quadrant 3); cells that were positive to Annexin-V were early apoptotic (Quadrant 4); cells positive both Annexin V and PI were late apoptotic (Quadrant 2); and cells that only stained with PI were necrotic or dead (Quadrant 1). ASE with an indicative concentration was treated against HeLa cells for 24 h. The distribution of normal healthy, early apoptotic, late apoptotic, and necrotic cell populations was represented in a bar diagram. Means sharing different letters differ significantly from each other at *p* ≤ 0.05.9. Effect of ASE on Caspase 3, 7, and 10 activity of Hela cells. Different concentrations of fungal crude extracts were treated to cells for 48 h and caspase 3, 7, 10 activity was determined by Caspase 3, 7,10 Apoptosis fluorometric Assay Kit. Means sharing different letters differ significantly from each other at p ≤ 0.05.

ROS production, a primary signal for apoptosis and shown to be involved in the mitochondrial-driven apoptosis (Yang et al., 2016), was assesses by DCFH-DA staining. It is a ROS specific fluorescent redox probe which gets oxidized to DCF (dichlorofluorescein) in the presence of ROS (Kumari et al., 2018). Supplementary Figure S2, showed the percentage of ROS positive cells (26.96, 49.64, and 80.57%) after HeLa cells were treated with increasing concentration of ASE (10, 25, and 50 μg/ml). Cells without treatment showed 2.5–3% cells served as negative control, 800 mM H_2_O_2_ for 15 min showed 96.21% served as positive control, After ASE 50 μg/ml treatment for 48 h media was replaced with 5 mM N-acetyl cysteine (NAC) for 1 h showed 66.57% served as another control. In the overlay graph showed in Figure 8A, ASE showed an increase in intracellular ROS production in a dose-dependent manner. After treatment with NAC, a ROS scavenger, the ROS production by ASE (50 μg/ml) reduced from 80 to 66%. NAC is a common antioxidant used to assess the ROS generation capabilities and ROS related antiproliferative roles of cytotoxic drugs (Khan et al., 2020). The results indicated that ROS generation induced by ASE in the HeLa cells contributes to apoptosis. Generation of ROS and high oxidative stress is one of the mechanisms; many potent drugs including taxol employ to exhibit multiple genetic alterations to initiate apoptosis in tumor cells *via* ROS-dependent mitochondrial dysfunction (Trachootham et al., 2009; Wu et al., 2017).

### Mitochondrial Membrane Depolarization Was Observed After JC 1 Staining

It is well known that mitochondria, a primary source of ROS generation contributes toward the apoptosis of cancer cells (Kumari et al., 2018). Mitochondrial membrane depolarization was assessed by cyanine dye JC one which facilitates discrimination of polarized and depolarized mitochondria because formation of the red aggregates when concentrated in polarized mitochondria in response to their higher membrane potential than the normally green fluorescent dye (Perelman et al., 2012).

We tested the effect of ASE on the depolarization of mitochondrial membrane in HeLa cells (Supplementary Figure S3). In healthy cells with intact mitochondria, JC-1 molecules aggregates to form a polymer-emitting orange-red fluorescence, whereas, in cells with depolarized mitochondria, JC-1 monomers emits green fluorescence in the cytosol (Cossarizza and Salvioli, 2001). After HeLa cells were treated with ASE, a shift in mitochondria with JC-1 staining red to green fluorescence was observed in a concentration-dependent manner (Figure 8B). An increased in the mitochondrial depolarization up to 20.20, 33.16, and 42.41% were observed when HeLa cells were treated with 10, 25, and 50 μg/ml of ASE for 24 h, respectively in a dose-dependent manner. Control cells without ASE treatment showed 8–10%, cells treated with 2,4-Dinitrophenol (2,4-DNP) served as positive control showed 78.53% loss of mitochondrial membrane potential. This suggests ASE-induced mitochondrial depolarization. Excessive generation of ROS may trigger mitochondrial depolarization leading to the intrinsic mitochondria-mediated pathway of apoptosis. Dandan et al., 2016 have also demonstrated hesperetin-induced ROS production activating the mitochondrial-mediated apoptosis in esophageal cancer cells.

### Annexin V-FITC/PI Staining Showed Induction of Apoptosis After ASE Treatment in the HeLa Cells

To further evaluate the mechanism of ASE-mediated cell death in HeLa cells, phosphatidylserine (PS) externalization was measured using FITC-labeled annexin V (Koopman et al., 1994; Martin et al., 1995). PS externalization on the plasma membrane in apoptotic cells is an intermediate event, occurring after mitochondrial membrane depolarization but before DNA fragmentation during cellular apoptosis (Suzuki et al., 2013). Annexin-V FITC/propidium iodide–based flow cytometry was used to detect healthy cells, early, late apoptotic cells, or dead cells. Annexin V-FITC binds to phosphatidylserine (PS) present on the outer side of the cell membrane of apoptotic cells. In healthy cells, PS remains on cytosolic side of plasma membrane making the cells negative to Annexin V-FITC (Singh et al., 2016). We conducted this assay to investigate the apoptosis-inducing potential of the ASE in HeLa cells. As indicated by FACS analysis, HeLa cells treated with the low concentration (10 μg/ml) of ASE exhibited 12% of cells in early apoptosis stage with 3.5% in late apoptosis. As the concentration increased, more cells shifted to early and late apoptosis in a dose-dependent manner (Figure 8C). The mechanism of ASE-induced cell death was by apoptosis. Apoptosis is one of the most common cell death mechanisms implied by natural products to induce cell death in cancer cells (Diederich, 2020; Balusamy et al., 2020). Endophytic *Aspergillus* sp. isolated from diverse host plants and related hosts have also shown apoptosis-mediated cell death on multiple cancer cell lines (Yao et al., 2016; Uzma et al., 2018).

### Caspase 3, 7, and 10 Fluorometric Assay Indicated Apoptotic Cell Death in HeLa Cells After Treatment With ASE

**FIGURE 9:**
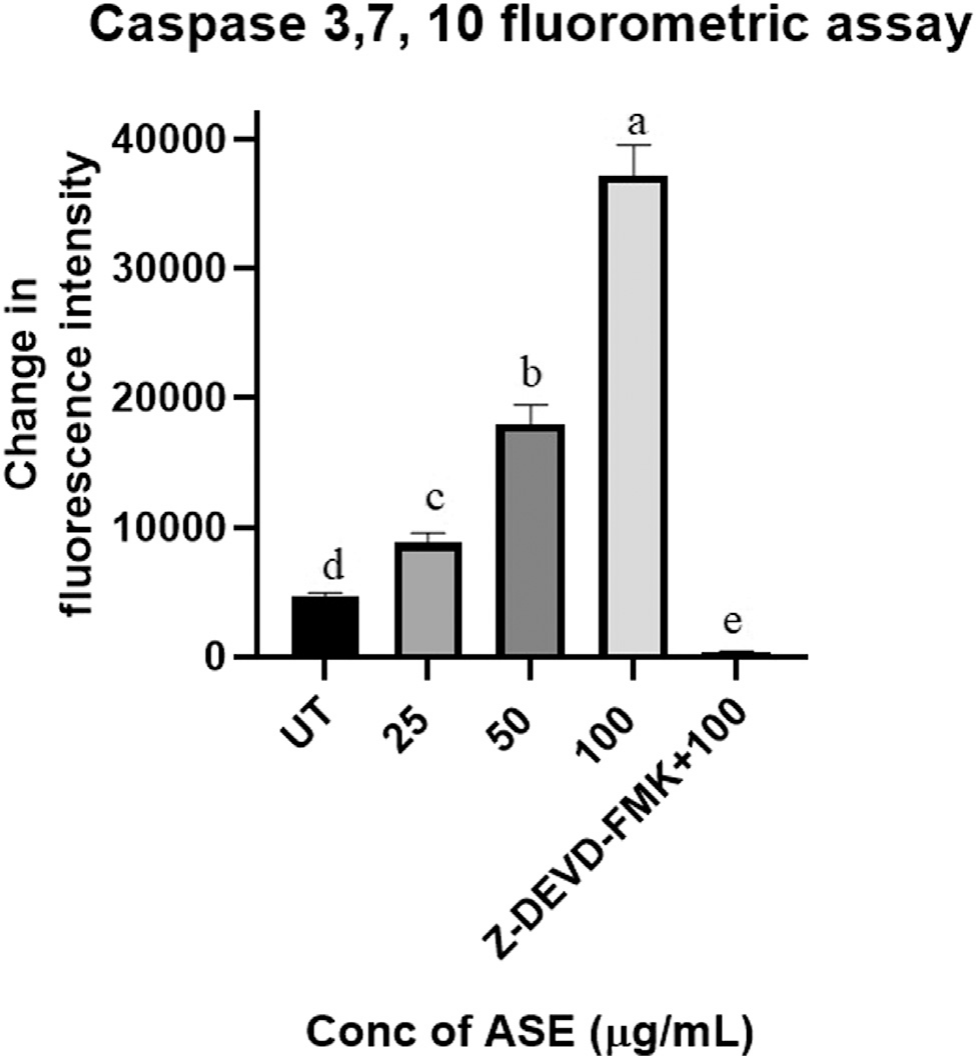
Effect of ASE on caspase 3, 7, and 10 activity of Hela cells. Different concentrations of fungal crude extracts were treated to cells for 48 h, and caspase 3, 7, and 10 activity was determined by caspase 3, 7, and 10 Apoptosis Fluorometric Assay Kit. Means sharing different letters differ significantly from each other at *p* ≤ 0.05.

Upregulation of Caspase 3, 7, and 10 proteins demonstrates the progression of apoptosis, making them the key indicators of this phenomenon (Brentnall et al., 2013). Activation of caspase three and seven are the prime events in apoptosis which initiates a cascade of reaction, leading to programmed cell death in cancer cells (McComb et al., 2019). The results demonstrated the dosage-dependent increase in fluorescence intensity, indicating activation of the caspase pathway in HeLa cells after treatment with different concentrations of ASE (Figure 9). Further, when apoptosis inhibitor Z-VAD-FMK was added to the cells treated with 100 μg/ml of ASE, a significant decrease in caspase activity was observed (Figure 9). The results clearly indicated the progression of cell death in treated HeLa cells by apoptosis.

## Conclusion

Resistance to cancer cells is steadily increasing, and many clinically used anticancer drugs are no longer effective. There is a high demand for innovative lead compounds to develop novel drugs for the treatment of cancer. Marine algal–derived endophytic fungi attract attention as a source of potential anticancer compounds. In the current study, cytotoxicity studies of crude extracts of endophytic fungi isolated from seaweed *S. muticum* showed that they possess cytotoxic compounds. In most of the cancer cell types, the arrest in cell cycle progression induced apoptosis. Our experiments demonstrated that the ethyl acetate extract of *Aspergillus* sp. arrested HeLa cell line at the G2/M stage of cell cycle progression, leading to apoptosis in human cervical cancer cells. The induction of apoptosis was supported by ROS production, MMP depolarization, phosphatidylserine (PS) externalization, and activation of the caspase pathway (Scheme 1). Further work is needed on the bioassay-guided purification of the crude extract to determine the structure of the specific compounds responsible for the observed bioactivities.

## Data Availability

The datasets presented in this study can be found in online repositories. The names of the repository/repositories and accession number(s) can be found in the article/Supplementary Material.
