# Supplementary material for: Optimization of PhysicoChemical Parameters for Production of Cytotoxic Secondary Metabolites and Apoptosis Induction Activities in the Culture Extract of a Marine Algal–Derived Endophytic Fungus Aspergillus sp
Source: Front Pharmacol. 2021 Apr 26;12:542891. doi: 10.3389/fphar.2021.542891 (PMC8108993; doi:10.3389/fphar.2021.542891)
Supplement: Supplementary file 1 [file datasheet1.docx]

**Supporting Information:**

**Optimization of physico-chemical parameters for production of cytotoxic secondary metabolites and apoptosis induction activities in the culture extract of a marine algal-derived endophytic fungus *Aspergillus* sp.**

**Sidhartha Taritla,^1^ Madhuree Kumari,^1^ Siya Kamat,^1^ Sarita G. Bhat,^2^ C. Jayabaskaran^1^***

*^1^Department of Biochemistry, Indian Institute of Science, Bangalore-560012, India*

*^2^Department of Biotechnology, Cochin University of Science and Technology, Kochi, Kerala 682022, India*

***Corresponding author:**

Prof. C. Jayabaskaran,

Department of Biochemistry,

Indian Institute of Science, Bangalore-560012,

India,

Tel: +91-80-22932482; Fax: +91-80-23600814;

E-mail: [cjb@iisc.ac.in](mailto:cjb@iisc.ac.in)

**Table S1: Different media used for the optimization of fungal anticancer secondary metabolites production**

| **Liquid Media** | **pH** | **Composition (g/L)** |
| --- | --- | --- |
| Gauce medium | 7.4 | Soluble starch 20; NaCl 0.5; KNO_3_ 1; K_2_HPO_4_.3H_2_O 0.5; MgSO_4_.7H_2_O 0.5; FeSO_4_.7H_2_O 0.01 |
| Malt Extract Broth (MEB) | 7.0 | Malt extract 20; Glucose 20; Peptone 1 |
| Potato Dextrose Broth (PDB) | 6.0 | Potato infusion 4; Dextrose 20 |
| Sabourard Broth (SDB) | 5.6 | Tryptone 10; Glucose 40 |
| Yeast Malt Extract Broth (YME) | 6.2 | Malt extract 10; yeast extract 4; MgSO_4_ 0.5; KH_2_PO_4_ 0.5 |
| Yeast Extract Phosphate Broth (YEP) | 6.0 | Yeast extract 1; KH_2_PO_4_ 6; NaH_2_PO_4_ 4; NH_4_OH 1 |
| Czapek Yeast Extract Broth (CZB) | 5.8 | NaNO_3_ 3; KH_2_PO_4_ 1; MgSO_4_.7H_2_O 0.5; KCl 0.5; FeSO_4_.7H_2_O 0.01; Glucose 30 |
| Goose and Tschessch Broth (GTB) | 7.0 | Peptone 2; Glucose 10; MgSO_4_.7H_2_O 0.5; KH_2_PO_4_ 0.5 |
| Leonine Broth (LEB) | 7.0 | Peptone 0.625; Maltose 6.25; Malt extract 6.25; KH_2_PO_4_ 1.25, MgSO_4_.7H_2_O 0.625 |


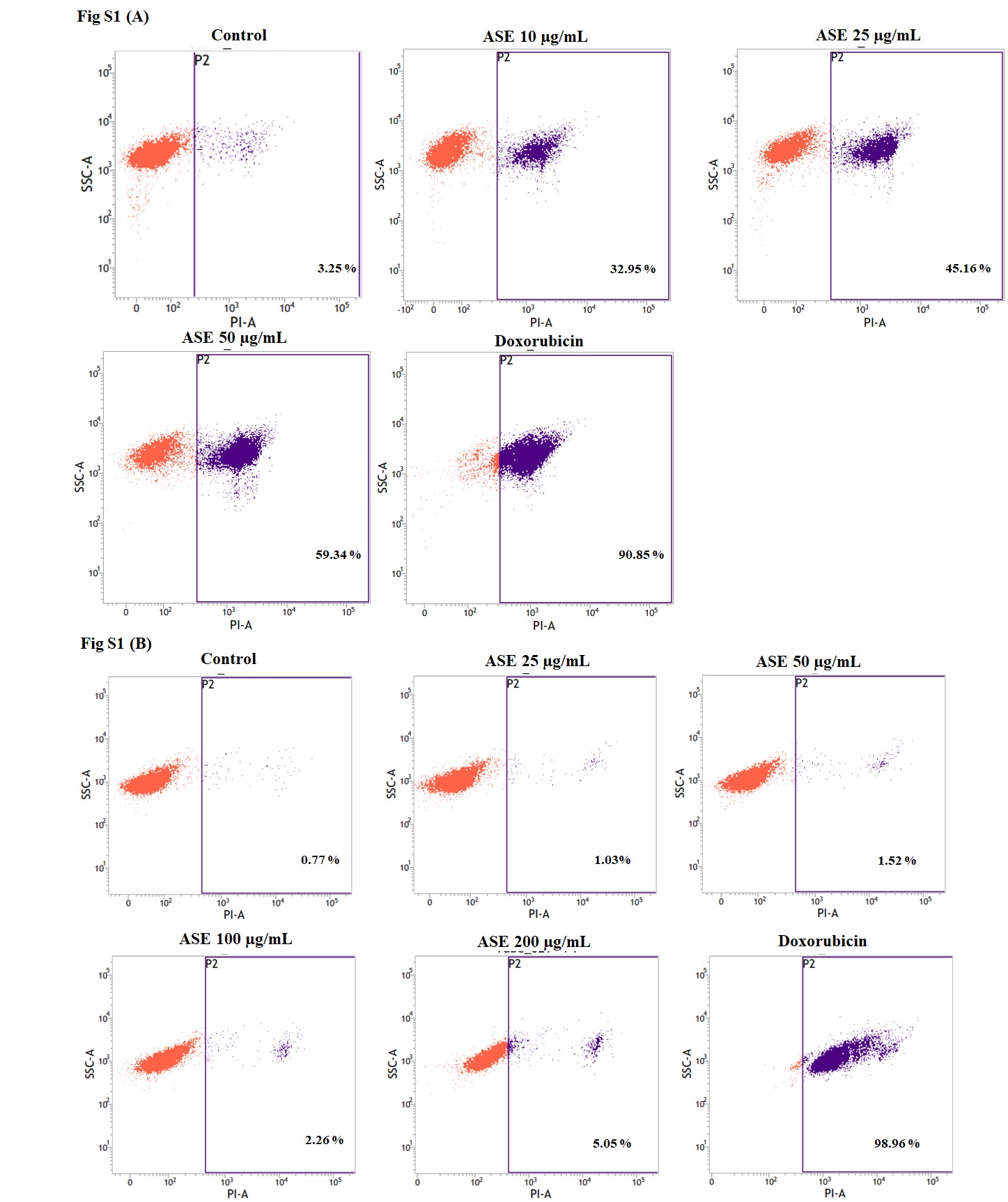


**Fig. S1:** Effects of ASE on cell viability on (A) HeLa cell line and (B) non-cancer CHO cell line in different concentrations by PI Live/Dead staining.


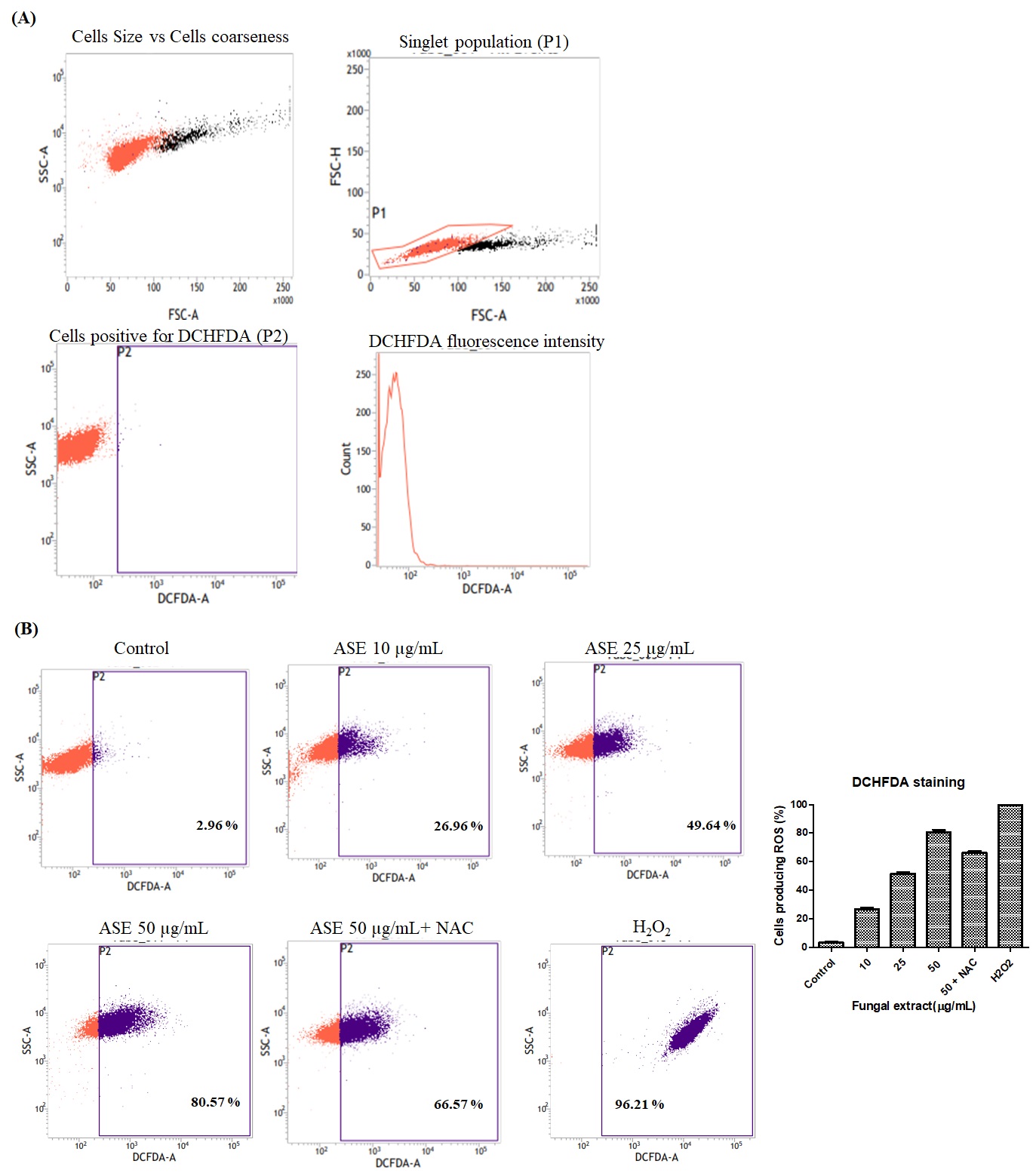


**Fig. S2.** (A) Gating parameters used for estimation of ROS produced by HeLa cells under different treatments of ASE (B) Effects of ASE on production of Reactive Oxygen Species (ROS) in different concentrations by DCFDA staining by flow cytometry. Bar Graph represents percent of ROS produced in different concentrations of ASE and ROS produced in absence and presence of ROS inhibitor (NAC) at 50 µg/mL of ASE.


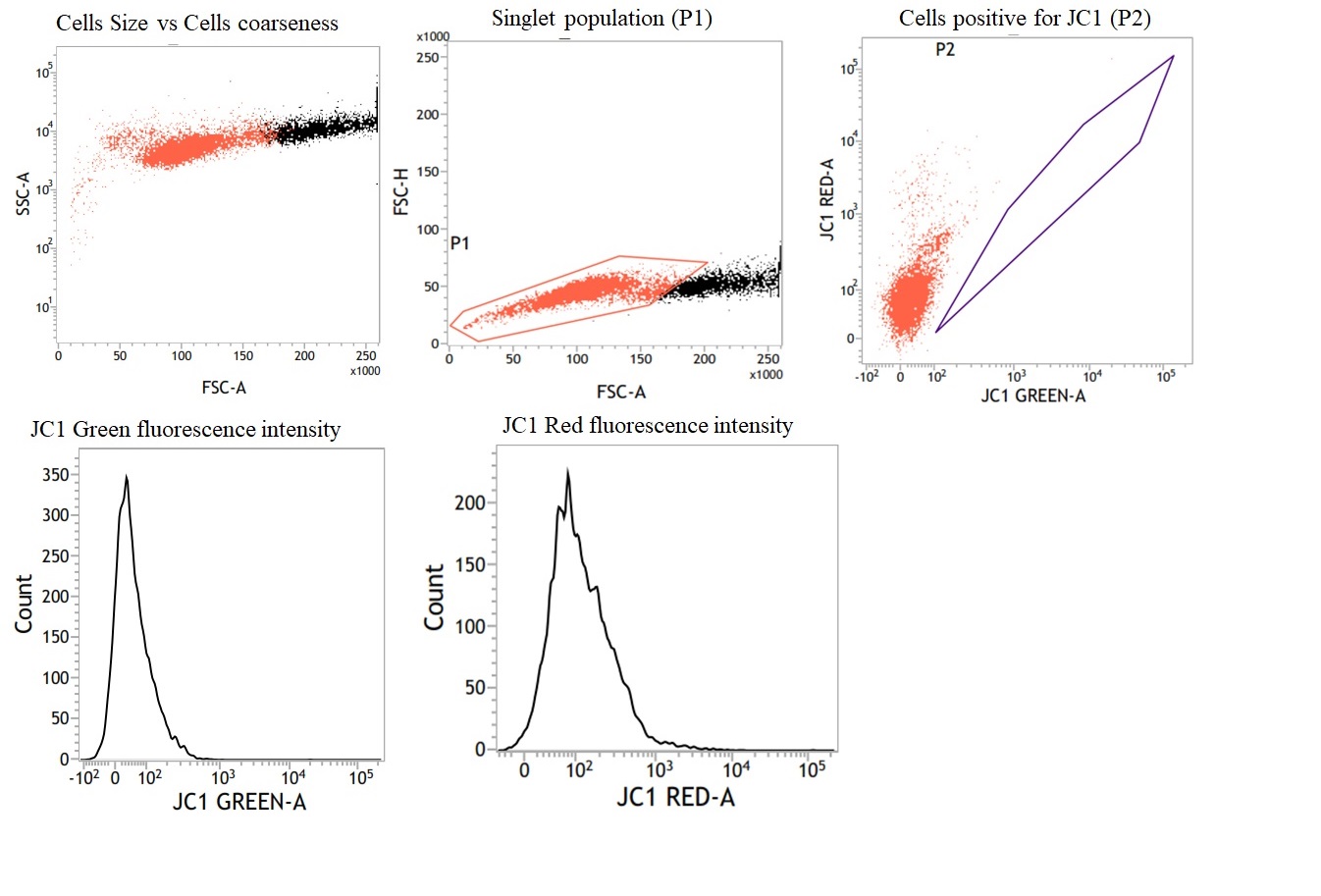


**Fig. S3.** Gating parameters used for estimation of loss in mitochondrial potential (MMP) by JC-1 staining by flow cytometry.
